# TpiA is a Key Metabolic Enzyme That Affects Virulence and Resistance to Aminoglycoside Antibiotics through CrcZ in Pseudomonas aeruginosa

**DOI:** 10.1128/mBio.02079-19

**Published:** 2020-01-07

**Authors:** Yushan Xia, Dan Wang, Xiaolei Pan, Bin Xia, Yuding Weng, Yuqing Long, Huan Ren, Jingyi Zhou, Yongxin Jin, Fang Bai, Zhihui Cheng, Shouguang Jin, Weihui Wu

**Affiliations:** a State Key Laboratory of Medicinal Chemical Biology, Key Laboratory of Molecular Microbiology and Technology of the Ministry of Education, Department of Microbiology, College of Life Sciences, Nankai University, Tianjin, China; b Department of Molecular Genetics and Microbiology, College of Medicine, University of Florida, Gainesville, Florida, USA; Inserm UMR-S P3Cell and CHU Reims; Harvard Medical School

**Keywords:** *Pseudomonas aeruginosa*, antibiotic resistance, carbon catabolite repression, triosephosphate isomerase, virulence

## Abstract

Carbon metabolism plays an essential role in bacterial pathogenesis and susceptibility to antibiotics. In Pseudomonas aeruginosa, Crc, Hfq, and a small RNA, CrcZ, are central regulators of carbon metabolism. By screening mutants of genes involved in carbon metabolism, we found that mutation of the *tpiA* gene reduces the expression of the type III secretion system (T3SS) and bacterial resistance to aminoglycoside antibiotics. TpiA is a triosephosphate isomerase that reversibly converts glyceraldehyde 3-phosphate to dihydroxyacetone phosphate, a key step connecting glucose metabolism with glycerol and phospholipid metabolisms. We found that mutation of the *tpiA* gene enhances the bacterial carbon metabolism, respiration, and oxidative phosphorylation, which increases the membrane potential and promotes the uptake of aminoglycoside antibiotics. Further studies revealed that the level of CrcZ is increased in the *tpiA* mutant due to enhanced stability. Mutation of the *crcZ* gene in the *tpiA* mutant background restored the expression of the T3SS genes and the bacterial resistance to aminoglycoside antibiotics. Overall, this study reveals an essential role of TpiA in the metabolism, virulence, and antibiotic resistance in P. aeruginosa.

## Abstract (precis)

The increase in bacterial resistance against antibiotics imposes a severe threat to public health. It is urgent to identify new drug targets and develop novel antimicrobials. Metabolic homeostasis of bacteria plays an essential role in their virulence and resistance to antibiotics. Recent studies demonstrated that antibiotic efficacies can be improved by modulating the bacterial metabolism. Pseudomonas aeruginosa is an important opportunistic human pathogen that causes various infections. The bacterium is intrinsically resistant to antibiotics. In this study, we provide clear evidence that TpiA (triosephosphate isomerase) plays an essential role in the metabolism of P. aeruginosa and influences bacterial virulence and antibiotic resistance. The significance of this work is in identifying a key enzyme in the metabolic network, which will provide clues as to the development of novel treatment strategies against infections caused by P. aeruginosa.

## INTRODUCTION

Pseudomonas aeruginosa is a ubiquitous opportunistic pathogen that causes a wide range of infections in humans, particularly in burn, immunodeficiency, and cystic fibrosis patients (1). P. aeruginosa harbors an arsenal of virulence factors that enable it to cause acute and chronic infections. The type III secretion system (T3SS) is a syringe-like machinery that directly injects effector proteins into host cells, leading to an alteration of cellular function or cell death (2). The T3SS plays an essential role in acute infections caused by P. aeruginosa (3). Chronic infections caused by P. aeruginosa are usually accompanied by biofilm formation, in which the bacteria are protected from host immune cells and antimicrobial substances by the extracellular matrix (4, 5). Besides biofilm formation, a variety of mechanisms harbored by P. aeruginosa contribute to its antibiotic resistance, including antibiotic modification/hydrolysis enzymes, low membrane permeability, and multidrug efflux systems (6, 7).

Successful colonization requires a bacterium to quickly adjust its metabolism to adapt to the *in vivo* host environment. P. aeruginosa possesses complex metabolic pathways enabling it to utilize various carbon sources. Carbon catabolite repression (CCR) optimizes the bacterial metabolism and improves its ability to thrive in natural habitats (8). Hfq, Crc, and a small RNA, CrcZ, play pivotal roles in CCR in P. aeruginosa. Crc forms a complex with Hfq, which directly represses translation by binding to target mRNAs (9). In the presence of less-preferred carbon sources, a CbrA/CbrB two-component system activates the transcription of CrcZ (10, 11). CrcZ then binds to and sequesters Hfq, which in turn abrogates Hfq-mediated translational repression (12). Besides regulating metabolic genes, CCR also controls bacterial virulence and antibiotic resistance. For instance, a mutation of *crc* reduces the expression of *exsA*, the central regulator of the T3SS (13). The quorum sensing (QS) signal molecule synthetase RhlI is a target of the Lon protease. Crc represses the translation of *lon*, thus stabilizing the RhlI and promoting the *rhl* QS pathway (14). Hfq has also been shown to regulate biofilm formation and contribute to bacterial resistance to gentamicin, fosfomycin, and tetracycline (15, 16). Recent studies demonstrated that carbon sources and cellular respiration influence bacterial susceptibility to antibiotics (17, 18). Meylan et al. reported that fumarate increases bacterial cellular respiration and proton motive force (PMF) by stimulating the tricarboxylic acid (TCA) cycle in P. aeruginosa, which promotes the uptake of tobramycin. In contrast, glyoxylate decreases bacterial susceptibility to tobramycin by promoting the glyoxylate shunt, which represses cellular respiration (19). Therefore, perturbation of the metabolic network influences bacterial virulence and antibiotic resistance.

Here, we examined the cytotoxicity and antibiotic resistance of P. aeruginosa strains with mutations in the carbon metabolism genes. We found that mutation of the triosephosphate isomerase gene *tpiA* reduced the bacterial virulence and resistance to aminoglycoside antibiotics. Further studies revealed a critical regulatory role of CrcZ in the *tpiA* mutant.

## RESULTS

### Identification of carbon metabolic genes that affect bacterial cytotoxicity and antibiotic resistance.

**FIG 1:**
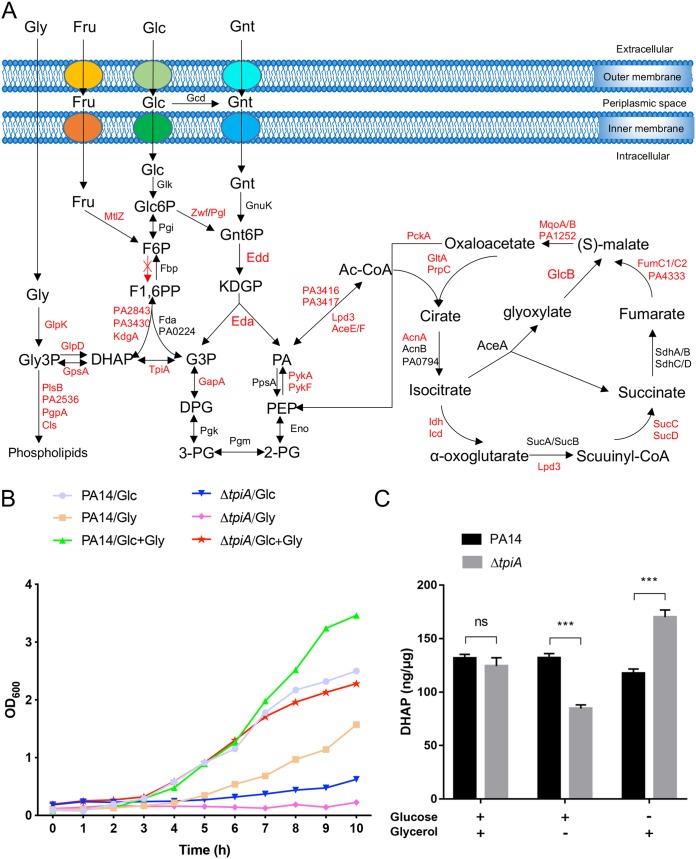
TpiA serves as a critical interlink between glycolysis and glycerol metabolism and phospholipid synthesis. (A) The glycolysis and tricarboxylic acid (TCA) cycle in P. aeruginosa. Most *Pseudomonas* species lack the phosphofructokinase that transforms fructose-6-phosphate (F6P) into fructose-1,6-biphosphate (F1,6PP) (highlighted with a crossed-out red arrow). Therefore, G3P and DHAP are not directly derived from glucose through the classical Embden-Meyerhof-Parnas (EMP) pathway, but rather from the ED pathway. The proteins labeled in red indicate that mutants of the corresponding genes were tested for cytotoxicity and antibiotic resistance. Glc, glucose; Gnt, gluconate; Fru, fructose; Glc6P, glucose-6-phosphate; Gnt6P, 6-phosphogluconate; KDPG, 2-keto-3-deoxy-6-phosphogluconate; G3P, glyceraldehyde-3-phosphate; DPG, 1,3-diphosphoglycerate; 3-PG, 3-phosphoglycerate; 2-PG, 2-phosphoglycerate; PEP, phosphoenolpyruvate; PA, pyruvate; DHAP, dihydroacetone phosphate; Ac-CoA, acetyl-coenzyme A; Gly, glycerol; Gly3P, glycerol-3-phosphate. (B) PA14 and the Δ*tpiA* mutant were grown overnight in LB. The bacteria were washed with PBS, and the same amount of the bacteria was inoculated in M9 medium with 40 mM glucose or 80 mM glycerol as the sole carbon source or containing both of the carbon sources (20 mM glucose and 40 mM glycerol). Bacterial growth was monitored by measuring the OD_600_ every hour for 10 h. (C) Bacterial intracellular DHAP levels. PA14 and the Δ*tpiA* mutant were grown in M9 medium with both 20 mM glucose and 40 mM glycerol to an OD_600_ of 1 and then washed three times with PBS. The bacteria were resuspended in M9 with 40 mM glucose or 80 mM glycerol as the sole carbon source and cultured for 1 h. The intracellular concentrations of DHAP were determined by a high-sensitivity dihydroxyacetone phosphate assay kit. The total protein levels were used as the internal controls. Data represent the mean ± standard deviation of the results from three samples. ns, not significant; ***, *P* < 0.001 by Student's *t* test.

To understand the influences of carbon metabolic genes on the virulence and antibiotic resistance of P. aeruginosa, we screened all of the available mutants from the PA14 transposon insertion mutant library (20), including genes involved in the TCA cycle, glycolysis, and gluconeogenesis (Fig. 1A). Among the 40 tested strains, mutations in the *aceE*, *aceF*, *tpiA*, and *gpsA* genes displayed reduced cytotoxicity toward the human alveolar epithelial cell A549, whereas the *gltA* and *PA2843* mutants displayed enhanced cytotoxicity (see Fig. S1 in the supplemental material). Meanwhile, mutants of *gltA*, *fumC1*, *pykF*, and *lpd3* were less susceptible to tobramycin, while the *zwf* and *tpiA* mutants were more susceptible to tobramycin (Table S1).

10.1128/mBio.02079-19.1 FIG S1 Cytotoxicity of strains with mutations in carbon metabolism-related genes. A549 cells were infected with indicated strains at a multiplicity of infection (MOI) of 50 for 3 h. The relative cytotoxicity was determined using an LDH release assay. Data represent the mean ± standard deviation of the results from three samples. **, *P* < 0.01; ***, *P* < 0.001 compared to PA14 by Student’s *t* test. Download FIG S1, PDF file, 0.1 MB. Copyright © 2020 Xia et al. 2020 Xia et al. This content is distributed under the terms of the Creative Commons Attribution 4.0 International license.

10.1128/mBio.02079-19.5 TABLE S1 Bacterial resistance levels to tobramycin in Mueller-Hinton broth. Download Table S1, DOCX file, 0.1 MB. Copyright © 2020 Xia et al. 2020 Xia et al. This content is distributed under the terms of the Creative Commons Attribution 4.0 International license.

Among all of the mutants tested, the *tpiA* mutant displayed simultaneous reduction in bacterial cytotoxicity and increased susceptibility to tobramycin to the greatest extent. The *tpiA* gene encodes a triosephosphate isomerase that catalyzes the reversible conversion from glyceraldehyde-3-phosphate (G3P) to dihydroxyacetone phosphate (DHAP). In P. aeruginosa, glucose is metabolized through the Entner-Doudoroff (ED) pathway via 6-phosphogluconate (Gnt6P) that is then dehydrogenated and turned into pyruvate and G3P (21, 22). Since P. aeruginosa lacks phosphofructokinase, it cannot transform fructose-6-phosphate into fructose-1,6-biphosphate. Thus, the TpiA-mediated conversion between G3P and DHAP serves as a critical link between glycolysis/gluconeogenesis and glycerol metabolism/phospholipid synthesis (23). Indeed, a severe growth defect was observed when a Δ*tpiA* mutant was cultured in a M9 medium with glucose or glycerol as the sole carbon source. When glycerol was the sole carbon source, the Δ*tpiA* mutant displayed no obvious growth after 10 h. When glucose was the sole carbon source, the maximum growth rates (MGR) of the Δ*tpiA* mutant and the wild-type PA14 were 0.03/h and 0.29/h, respectively (Fig. 1B). However, simultaneous supplementation of both glucose and glycerol enabled the growth of the Δ*tpiA* mutant (Fig. 1B), resulting in the similar MGR in the Δ*tpiA* mutant and the wild-type PA14 (0.33/h and 0.32/h, respectively).

To further verify the function of TpiA, wild-type PA14 and the Δ*tpiA* mutant were grown in M9 medium with both glucose and glycerol as the carbon sources to an optical density at 600 nm (OD_600_) of 1.0, which resulted in similar intracellular concentrations of DHAP in the two strains (Fig. 1C). However, the intracellular concentration of DHAP was lower in the Δ*tpiA* mutant 1 h after the bacteria were switched into the medium with glucose as the sole carbon source, and the reverse was the case when glycerol was the sole carbon source (Fig. 1C). In combination, these results demonstrated a critical role of TpiA in the carbon metabolism of P. aeruginosa.

### The type III secretion system is defective in the *tpiA* mutant.

**FIG 2:**
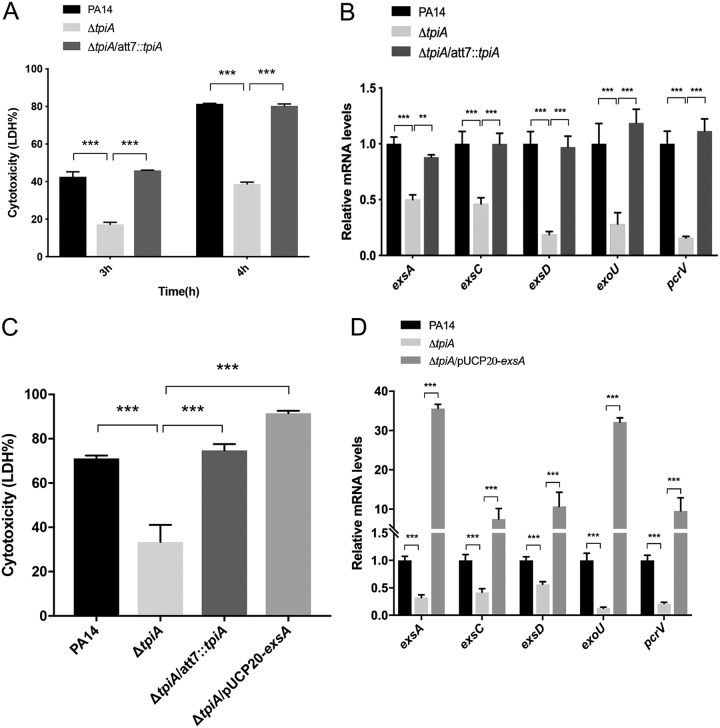
TpiA influences the T3SS. (A) Cytotoxicity of wild-type PA14, the Δ*tpiA* mutant, and the complemented strain. A549 cells were infected with the indicated strains at a multiplicity of infection (MOI) of 50 for 3 h or 4 h. The relative cytotoxicity was determined by the LDH release assay. (B) Wild-type PA14, the Δ*tpiA* mutant, and the complemented strain were grown in LB with 5 mM EGTA for 4 h. The relative mRNA levels of the T3SS genes were determined by real-time PCR. The 30S ribosomal protein gene *rpsL* was used as an internal control. (C) Cytotoxicity of the strains of wild-type PA14, the Δ*tpiA* mutant, the complemented strain, and the Δ*tpiA* mutant overexpressing *exsA*. A549 cells were infected with the bacteria with an MOI of 50 for 4 h. The relative cytotoxicity was determined by the LDH release assay. (D) The indicated bacterial strains were grown in LB with 5 mM EGTA for 4 h. The relative mRNA levels of the T3SS genes were determined by real-time PCR. The 30S ribosomal protein gene *rpsL* was used as an internal control. Data represent the mean ± standard deviation of the results from three samples. *, *P* < 0.05; **, *P* < 0.01; ***, *P* < 0.001 by Student's *t* test.

Similar to the *tpiA*::Tn mutant, the Δ*tpiA* mutant displayed defective cytotoxicity, which was restored by complementation with the *tpiA* gene (Fig. 2A). Since the T3SS plays a critical role in the cytotoxicity of P. aeruginosa, we examined the expression levels of several T3SS genes, including the regulatory genes *exsA*, *exsC*, and *exsD*, the needle tip component gene *pcrV*, and the effector gene *exoU*. The *tpiA* mutant showed reduced expression of all of those genes, which was restored by complementation with the *tpiA* gene (Fig. 2B). Overexpression of the T3SS master regulatory gene *exsA* restored the mRNA levels of the *exsC*, *exsD*, *pcrV*, and *exoU* genes and the cytotoxicity of the Δ*tpiA* mutant (Fig. 2C and D). These results suggested that TpiA affects the expression of the T3SS genes.

### Mutation of the *tpiA* gene increases bacterial susceptibility to aminoglycoside antibiotics.

We then tested the role of TpiA in the bacterial resistance to various classes of antibiotics, including aminoglycosides, fluoroquinolones, tetracycline, polymyxin B, and β-lactams. Mutation of the *tpiA* gene did not affect the bacterial resistance to ciproﬂoxacin, ofloxacin, tetracycline, or carbenicillin. However, the Δ*tpiA* mutant exhibited increased susceptibility to all aminoglycoside antibiotics, with an 8-fold decrease in the MICs of tobramycin and a 4-fold decrease in the MICs of gentamicin, streptomycin, neomycin, and amikacin. Also, the Δ*tpiA* mutant exhibited a 2-fold decrease in the MIC of polymyxin B. Complementation with the *tpiA* gene restored the resistance to these antibiotics (Table 1). Since TpiA is involved in carbon metabolism, it may affect the bacterial antibiotic resistance differently in different nutrient environments. Therefore, we determined the MICs of aminoglycoside antibiotics in an artificial sputum medium (ASM) that had been used to mimic the lung environment of cystic fibrosis patients (24, 25). The Δ*tpiA* mutant displayed a 4-fold decrease in the MICs of gentamicin, neomycin, and amikacin and 8-fold decreases in the MICs of tobramycin and streptomycin (Table 2).

**TABLE 1:** MICs of indicated strains in MHB

Strain	MIC (μg/ml)^*a*^										
	GEN	TOB	NEO	STR	AMK	TET	MEM	CAR	PMB	OFX	CIP
PA14	1	1	16	16	2	32	0.25	64	4	1	0.25
Δ*tpiA* mutant	0.25	0.125	4	4	0.5	32	0.25	64	2	1	0.25
Δ*tpiA*/att7::*tpiA*	1	1	16	16	2	32	0.25	64	4	1	0.25

a GEN, gentamicin; TOB, tobramycin; NEO, neomycin; STR, streptomycin; AMK, amikacin; TET, tetracycline; MEM, meropenem; CAR, carbenicillin; PMB, polymyxin B; OFX, ofloxacin; CIP, ciprofloxacin.

**TABLE 2:** MICs of the aminoglycoside antibiotics to indicated strains in ASM

Strain	MIC (μg/ml)^*a*^				
	GEN	TOB	NEO	STR	AMK
PA14	2	2	64	16	4
Δ*tpiA* mutant	0.5	0.25	16	4	0.5

a GEN, gentamicin; TOB, tobramycin; NEO, neomycin; STR, streptomycin; AMK, amikacin.

### Mutation of the *tpiA* gene increases the intracellular tobramycin level.

**FIG 3:**
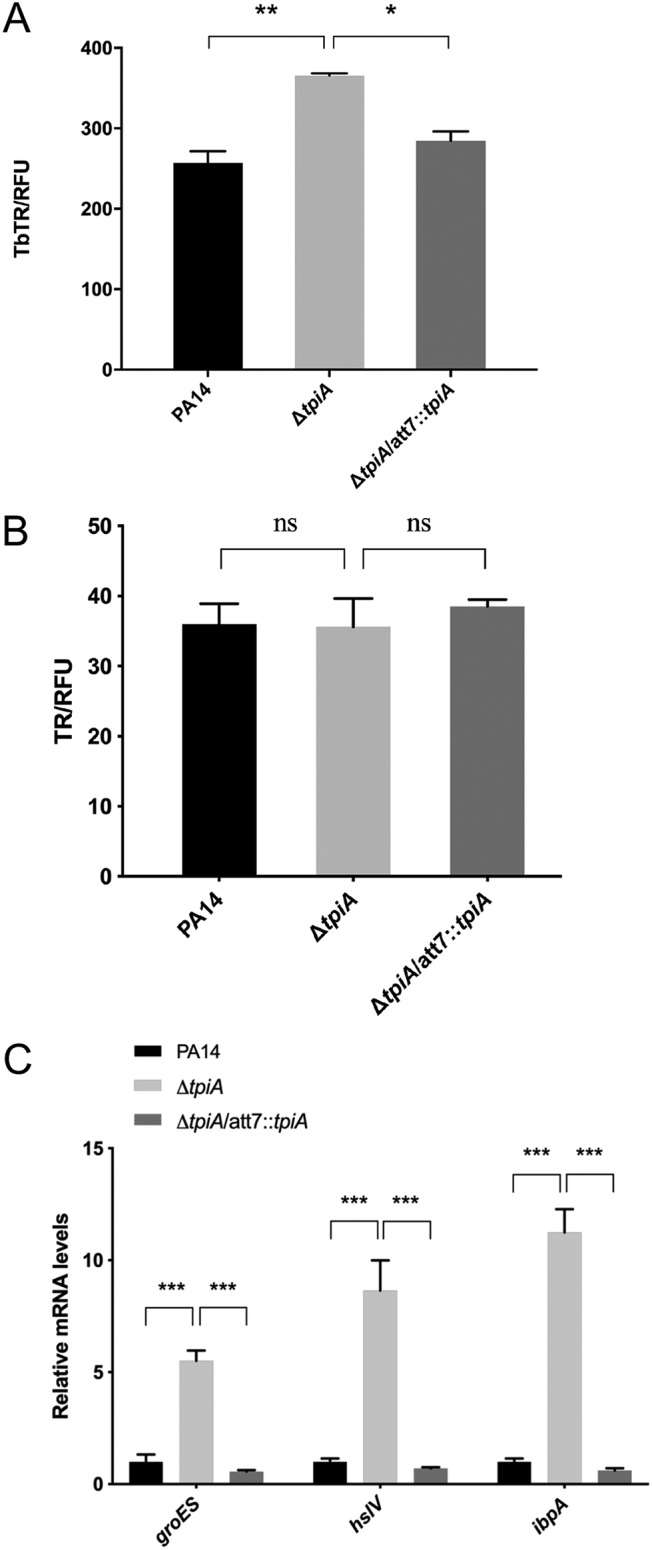
Mutation of *tpiA* increases the uptake of tobramycin. (A and B) Wild-type PA14, the Δ*tpiA* mutant, and the complemented strain were cultured in LB to an OD_600_ of 1, followed by incubation with 40 mg/liter tobramycin-Texas Red (TbTR) (A) or Texas Red (TR) (B) for 30 min. The bacteria were collected by centrifugation, washed three times with PBS, and then lysed in PBS by sonication. The Texas Red was excited at 595 nm, and the emission at 615 nm was measured with a luminometer. The relative fluorescence (measured in relative fluorescence units [RFU]) was normalized by the total protein amount. (C) The bacteria were treated with 0.1 mg/liter tobramycin for 30 min, followed by RNA isolation. The mRNA levels of *groES*, *hslV*, and *ibpA* were determined by real-time PCR. The 30S ribosomal protein gene *rpsL* was used as an internal control. Data represent the mean ± standard deviation of the results from three samples. *, *P* < 0.05; **, *P* < 0.01; ***, *P* < 0.001; ns, not significant by Student's *t* test.

Limiting intracellular drug levels is a major intrinsic resistance mechanism of P. aeruginosa against aminoglycoside antibiotics. Therefore, we measured tobramycin uptake by utilizing a Texas Red-labeled tobramycin (designated TbTR) (19). A larger amount of the TbTR was observed in the Δ*tpiA* mutant cells than in wild-type PA14 (Fig. 3A), whereas no difference was observed in the uptake of the free Texas Red (Fig. 3B). A previous study demonstrated that intracellular tobramycin activates the expression of genes involved in the heat shock response (26). Consistent with the TbTR uptake results, tobramycin treatment resulted in higher mRNA levels of the heat shock response genes, including *groES*, *ibpA*, and *hslV* in the Δ*tpiA* mutant, which were restored to the PA14 level by complementation with the *tpiA* gene (Fig. 3C). In combination, these results demonstrate increased tobramycin uptake by the Δ*tpiA* mutant.

### The increased uptake of tobramycin is due to respiratory enhancement in the Δ*tpiA* mutant.

The intracellular tobramycin level is largely influenced by efflux and uptake. The multidrug efflux system MexXY-OprM plays an important role in bacterial resistance to aminoglycoside antibiotics. Mutation or defective expression of *mexX* or *mexY* increases the bacterial susceptibility to aminoglycoside antibiotics (27, 28). Therefore, we examined whether mutation of *tpiA* affects the expression of *mexXY* in the presence or absence of tobramycin. The tobramycin was used at 0.1 mg/liter, at which no growth difference was observed between the wild-type PA14 and the Δ*tpiA* mutant (data not shown). The mRNA levels of *mexX*, *mexY*, and *oprM* were similar between wild-type PA14 and the Δ*tpiA* mutant in the presence or absence of tobramycin (Fig. S2).

10.1128/mBio.02079-19.2 FIG S2 Expression levels of the efflux pump genes in the Δ*tpiA* mutant. The bacteria were cultured in LB to an OD_600_ of 1 and then incubated with or without 0.1 mg/liter tobramycin for 30 min. The mRNA levels of indicated genes were determined by real-time PCR. The 30S ribosomal protein gene *rpsL* was used as an internal control. Data represent the mean ± standard deviation of the results from three samples. Download FIG S2, PDF file, 0.1 MB. Copyright © 2020 Xia et al. 2020 Xia et al. This content is distributed under the terms of the Creative Commons Attribution 4.0 International license.

**FIG 4:**
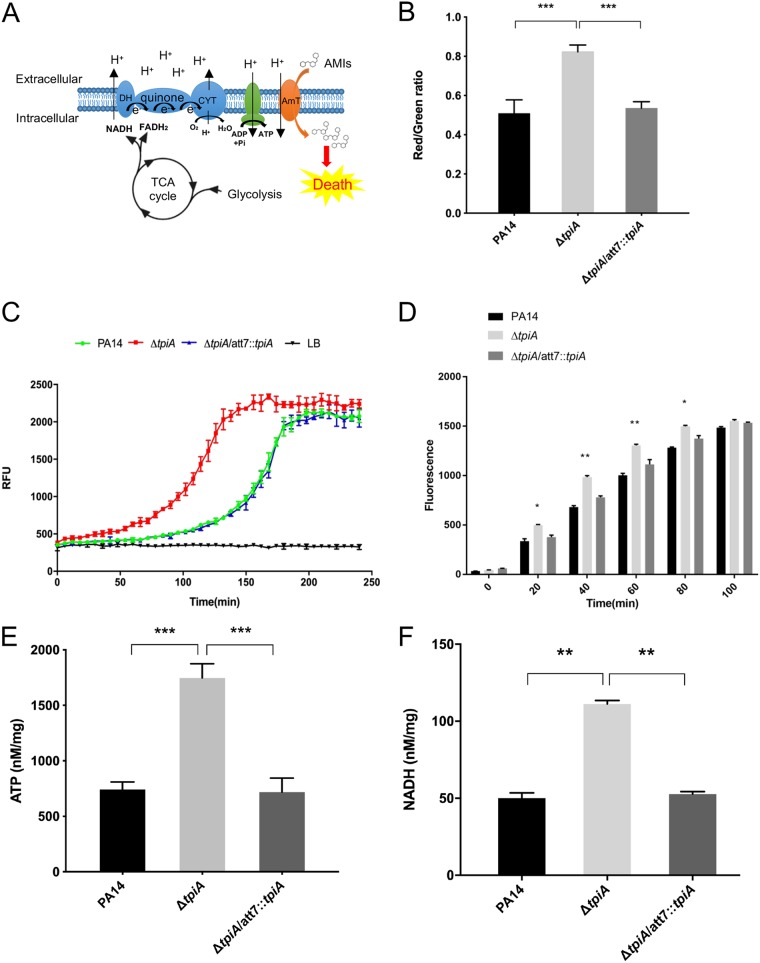
The increased uptake of tobramycin is due to respiratory enhancement in the *tpiA* mutant. (A) Schematic of the respiratory chain, glycolysis, and TCA cycle. DH, dehydrogenase; QE, quinone; CYT, cytochrome oxidase. Proton motive force (PMF) generated by the ETC drives aminoglycoside (19) uptake through a putative transporter (AmT). (B) Membrane potential was represented by the ratio of green to red fluorescence. The logarithmic-phase bacterial cells were incubated with DiOC_2_(3) for 1 h and then subjected to flow cytometry analysis. Data represent the mean ± standard deviation of the results from three samples. ***, *P* < 0.001 by Student's *t* test. (C) Bacterial ETC activity. The ETC activities were quantified by an extracellular oxygen consumption assay kit. A total of 1.5 × 10^5^ bacterial cells in fresh LB were mixed with 10 μl of the O_2_ consumption reagent in a black 96-well plate and sealed with 50 μl mineral oil to isolate the air. As the bacterial respires, oxygen is depleted in the surrounding environment, which is seen as an increase in phosphorescence signal. The fluorescence was measured every 5 min for 4 h. Data represent the mean ± standard deviation of the results from three samples. (D) Bacterial respiratory rates were quantified by alamarBlue, a nonfluorescent dye that emits pink fluorescence in the reduced state. A total of 2 × 10^8^ bacterial cells in fresh LB were mixed with 20 μl alamarBlue reaction solution in a black 96-well plate. The fluorescence was measured every 20 min for 2 h. Data represent the mean ± standard deviation of the results from three samples. (E) Intracellular ATP levels were determined using an enhanced ATP assay kit. Bacteria were cultured in LB at 37°C to an OD_600_ of 1. One and a half milliliters of the bacteria was collected and broken by ultrasound sonication in the lysis buffer. Measurement of the ATP level in the supernatant was performed according to the manufacturer’s instruction. Total proteins were quantified by bicinchoninic acid (BCA) analysis for normalization. Data represents the mean ± standard deviation from three samples. (F) Bacterial NADH levels were determined by an Amplite fluorimetric NAD/NADH ratio assay kit. Bacteria were cultured in LB medium at 37°C to an OD_600_ of 1. The bacteria were lysed in PBS buffer by sonication. Measurement of the NADH level in the supernatant was performed according to the manufacturer’s instruction. Total proteins were quantified by BCA analysis for normalization. Data represent the mean ± standard deviation of the results from three samples. **, *P* < 0.01; ***, *P* < 0.001 by Student's *t* test.

We then examined whether TpiA influences the uptake of tobramycin. Previous studies revealed that the proton motive force (PMF) generated by the electron transport chain (ETC) is required for the uptake of tobramycin (Fig. 4A) (29). We used the dye 3,3′-diethyloxacarbocyanine iodide [DiOC_2_(3)] to examine PMF-dependent membrane potential, which is represented by the ratio of red to green ﬂuorescence (19, 30). As shown in Fig. 4B, mutation of *tpiA* enhanced the membrane potential. By utilizing an extracellular oxygen consumption assay kit and alamarBlue (31), we found that mutation of *tpiA* enhanced the bacterial ETC activity and respiratory rate (Fig. 4C and D). In addition, the levels of ATP and NADH were also increased in the Δ*tpiA* mutant (Fig. 4E and F).

**FIG 5:**
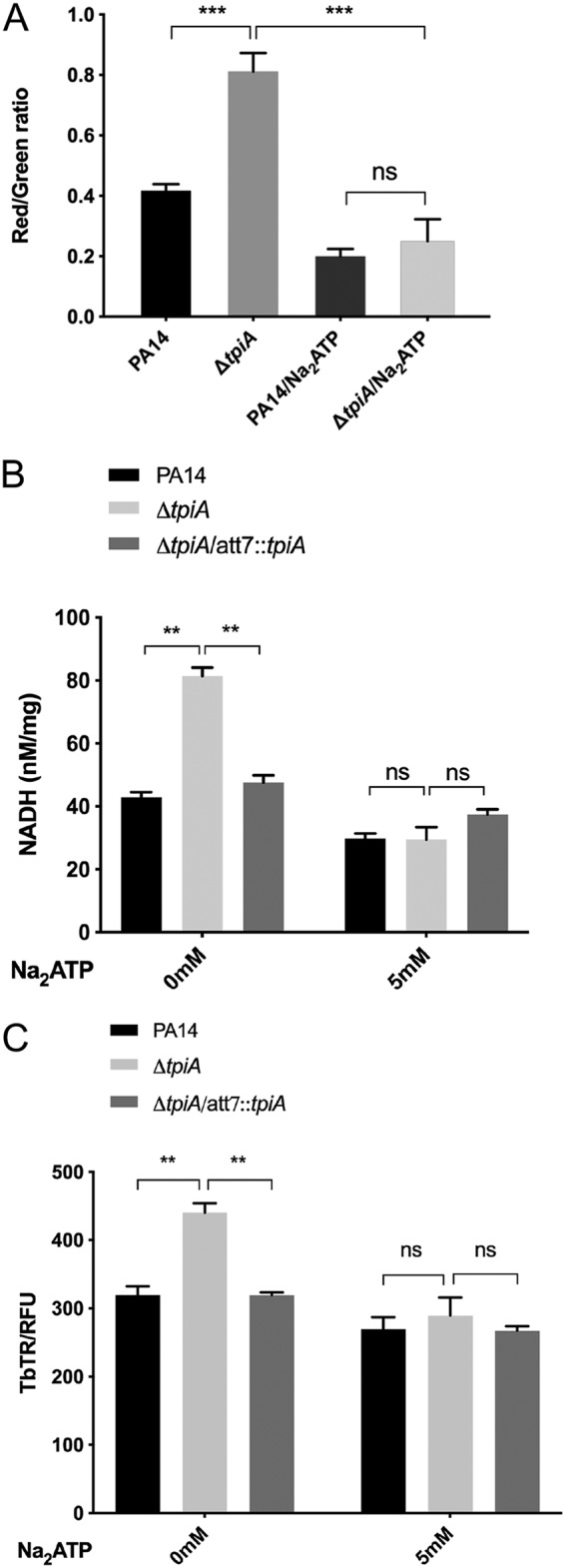
Inhibition of the TCA cycle restores the resistance of the Δ*tpiA* mutant to aminoglycoside antibiotics. (A) Effect of TCA inhibitor on membrane potential. PA14 and the Δ*tpiA* mutant were cultured in LB with or without 5 mM Na_2_ATP to an OD_600_ of 1. The bacteria were diluted to 10^6^ CFU/ml in PBS. Two microliters of 20 mM DiOC_2_(3) was added to 1.5 ml of the bacterial suspension and incubated for 1 h at 37°C in dark, followed by ﬂow cytometry analyses. (B) Effect of the TCA inhibitor on the NADH level. The bacteria were cultured in LB with or without 5 mM Na_2_ATP to an OD_600_ of 1. The bacterial cells were collected and lysed in PBS by sonication. Measurement of the NADH level in the supernatant was performed according to the manufacturer’s instruction. (C) Effect of the TCA inhibitor on tobramycin uptake. The bacteria were cultured in LB with or without 5 mM Na_2_ATP to an OD_600_ of 1 and then incubated with 40 mg/liter tobramycin-Texas Red (TbTR) for 30 min. The bacteria were collected by centrifugation and washed three times with PBS, followed by lysis in PBS by sonication. The fluorescence was measured with a luminometer. The relative fluorescence was normalized by the corresponding total protein concentration. Data represent the mean ± standard deviation of the results from three samples. **, *P* < 0.01; ***, *P* < 0.001 by Student's *t* test.

To determine whether the enhanced respiratory activity is the cause of increased susceptibility to tobramycin in the Δ*tpiA* mutant, we treated the bacterial cells with Na_2_ATP, an inhibitor of TCA cycle enzymes, including citrate synthase, isocitric dehydrogenase, and α-oxoglutarate dehydrogenase (32). In MHB, the Δ*tpiA* mutant grows slower than does the wild-type strain (Fig. S3A). Treatment with Na_2_ATP reduced the growth rates of both of the strains (Fig. S3A). In addition, treatment with Na_2_ATP reduced the membrane potential, intracellular NADH level, and uptake of tobramycin in both strains, diminishing the differences observed between the two strains without the Na_2_ATP treatment (Fig. 5A to C). Furthermore, the presence of Na_2_ATP increased the MICs of tobramycin for wild-type PA14 and the Δ*tpiA* mutant by 8- and 32-fold, respectively, which reduced the difference between the two strains from 8-fold to 2-fold (Table 3). Therefore, mutation of *tpiA* results in respiratory enhancement, which subsequently increases bacterial susceptibility to tobramycin.

**TABLE 3:** MICs of tobramycin against indicated strains in MHB with or without Na_2_ATP

Strain	MIC (μg/ml)		
	0 mM	2 mM	5 mM
PA14	1	2	8
Δ*tpiA* mutant	0.125	1	4

10.1128/mBio.02079-19.3 FIG S3 Growth curves of bacteria in different media. Overnight cultures of the indicated strains were diluted 1:100 into fresh LB. The bacterial growth was monitored by measuring the OD_600_ every hour for 10 hours. (A) The growth curves of indicated strains grown in MHB with or without 5mM Na_2_ATP. (B and C) The growth curves of indicated strains grown in MHB (B) and LB (C). Download FIG S3, PDF file, 0.2 MB. Copyright © 2020 Xia et al. 2020 Xia et al. This content is distributed under the terms of the Creative Commons Attribution 4.0 International license.

### Mutation of *tpiA* releases the carbon catabolite repression.

**FIG 6:**
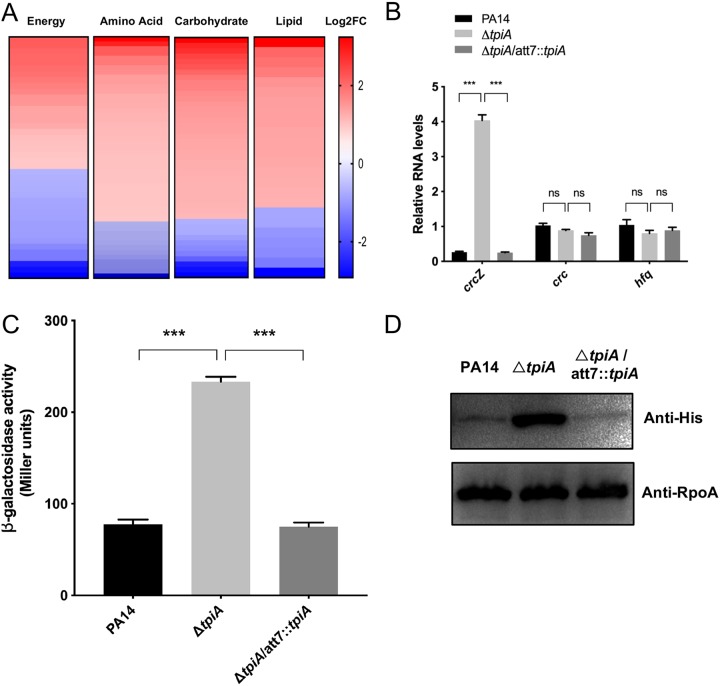
Mutation of the *tpiA* gene affects the expression of metabolic genes. (A) RNA sequencing (RNA-seq) results. Values are reported as log_2_ fold change (Log2FC) of the expression of energy metabolism, amino acid metabolism, carbohydrate metabolism, and lipid metabolism genes in the Δ*tpiA* mutant over wild-type PA14. (B) The bacteria were cultured in LB to an OD_600_ of 1. The RNA levels of *crc*, *hfq*, and *crcZ* were determined by real-time PCR. The 30S ribosomal protein gene *rpsL* was used as an internal control. Data represent the mean ± standard deviation of the results from three samples. ***, *P* < 0.001 by Student's *t* test. (C) Indicated strains containing the *amiE′*-*lacZ* translation fusion were cultured in LB with 1 mM isopropyl-β-d-thiogalactopyranoside (IPTG). When the OD_600_ reached 1, the bacteria were collected, followed by the β-galactosidase activity assay. Data represent the mean ± standard deviation of the results from three samples. ***, *P* < 0.001 by Student's *t* test. (D) Indicated strains containing the *amiE*-His translational fusion were cultured in LB with 0.2% l-arabinose. When the OD_600_ reached 1, the bacteria were collected. The AmiE-His levels were determined by Western blotting with RpoA as the loading control.

Previous studies demonstrate that bacterial respiratory activity is closely related to the expression of the ETC and central metabolic genes (19). To understand how TpiA affects bacterial respiration, we compared the transcriptomes of wild-type PA14 and the Δ*tpiA* mutant. A total 846 genes were differentially expressed in the Δ*tpiA* mutant. The GO and KEGG pathway enrichment analyses revealed that mutation of *tpiA* resulted in the upregulation of multiple carbon metabolism and ETC genes, including the aconitate hydratase gene *acnA*, fumarate hydratase gene *fumC2*, dihydrolipoamide dehydrogenase gene *lpd3* in the TCA cycle, the pyruvate dehydrogenase component subunit genes *PA3416* and *PA3417*, and the cytochrome *c* oxidase subunit genes *coxA*, *coxB*, *coIII*, *coiA*, and *PA4133* in oxidative phosphorylation (Fig. 6A and Table S2). In addition, multiple genes involved in amino acid and fatty acid metabolism were upregulated in the Δ*tpiA* mutant (Table S2). In P. aeruginosa, Crc, Hfq and a small RNA, CrcZ, play important roles in carbon catabolite repression (CCR) that globally regulates metabolic genes (9). The expression patterns of the above-described genes in the Δ*tpiA* mutant resemble those in a *crc* or *hfq* mutant (12, 33). We thus examined the expression levels of *crc*, *hfq*, and *crcZ* by real-time PCR. No significant change was observed in the expression of *crc* and *hfq*, whereas *crcZ* was upregulated in the Δ*tpiA* mutant (Fig. 6B), indicating a possible release of CCR. We then examined the translation efficiency of *amiE* (encoding the aliphatic amidase), which is inhibited by Crc and Hfq in CCR (11, 34). We constructed a translational fusion between a *lacZ* reporter gene and the CRC regulatory sequence of *amiE* (*amiE′*-*lacZ*) (11) as well as a C-terminal 6×His-tagged *amiE* (*amiE*-His), which are driven by an exogenous P*_tac_* promoter and P*_BAD_* promoter, respectively. The LacZ and *amiE*-His levels were higher in the Δ*tpiA* mutant than those in the wild-type PA14 and complemented strains (Fig. 6C and D), indicating a release of CCR.

10.1128/mBio.02079-19.6 TABLE S2 Expression levels of carbon metabolism-related genes. Download Table S2, DOCX file, 0.1 MB. Copyright © 2020 Xia et al. 2020 Xia et al. This content is distributed under the terms of the Creative Commons Attribution 4.0 International license.

### Mutation of the *tpiA* gene increases the stability of CrcZ.

**FIG 7:**
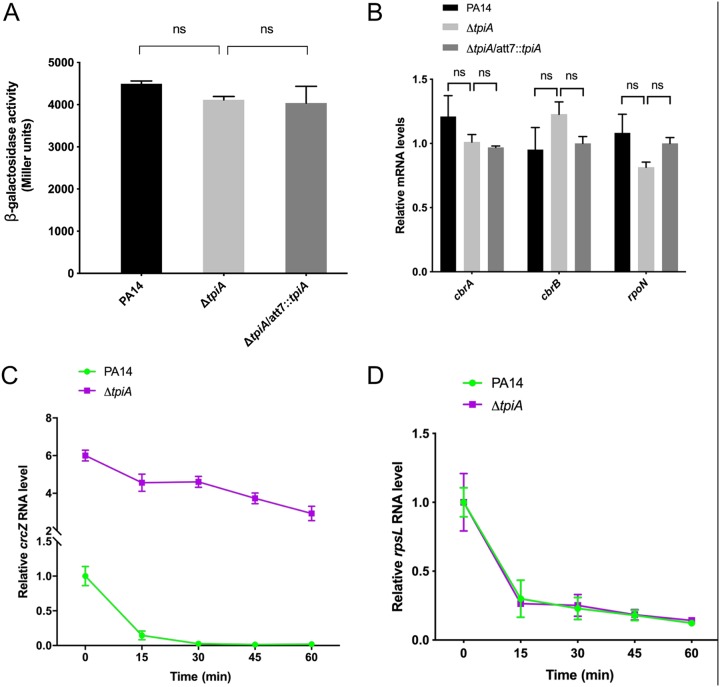
Mutation of the *tpiA* gene increases the stability of CrcZ. (A) PA14, the Δ*tpiA* mutant, and the complemented strain containing the P*_crcZ_*-*lacZ* transcriptional fusion were cultured in LB to an OD_600_ of 1. The bacteria were collected, followed by the β-galactosidase activity assay. (B) The indicated strains were cultured in LB to an OD_600_ of 1. The mRNA levels of *cbrA*, *cbrB*, and *rpoN* were determined by real-time PCR. The 30S ribosomal protein gene *rpsL* was used as an internal control. Data represent the mean ± standard deviation of the results from three samples. ns, not significant. (C and D) Degradation of CrcZ (C) and the *rpsL* mRNA (D) in wild-type PA14 and the Δ*tpiA* mutant. Bacterial cells were treated with rifampin, and at the indicated time points, the bacteria were collected and mixed with equal numbers of *gfp*-expressing E. coli cells. Total RNA was isolated, and the relative RNA levels were determined by real-time PCR. The *gfp* RNA level in each sample was used as the internal control for normalization.

To explore the mechanism of the CrcZ upregulation, we constructed a transcriptional fusion between the *lacZ* reporter gene and the promoter of *crcZ* (P*_crcZ_*-*lacZ*). The LacZ levels were similar in wild-type PA14 and the Δ*tpiA* mutant (Fig. 7A). Consistently, no significant difference was observed in the known transcriptional regulatory genes of the *crcZ*, including *rpoN*, *cbrA*, and *cbrB* (Fig. 7B) (8). We then examined stability of CrcZ. After blockage of RNA transcription by rifampin, the CrcZ level dropped quickly in wild-type PA14, whereas a slower reduction was observed in the Δ*tpiA* mutant (Fig. 7C). Meanwhile, the rRNA RpsL level was reduced at a similar rate in the two strains (Fig. 7D). These results suggested that the higher level of CrcZ in the Δ*tpiA* mutant is likely due to its increased stability.

### The increased CrcZ level in the Δ*tpiA* mutant contributes to its hypersusceptibility to aminoglycoside antibiotics.

**FIG 8:**
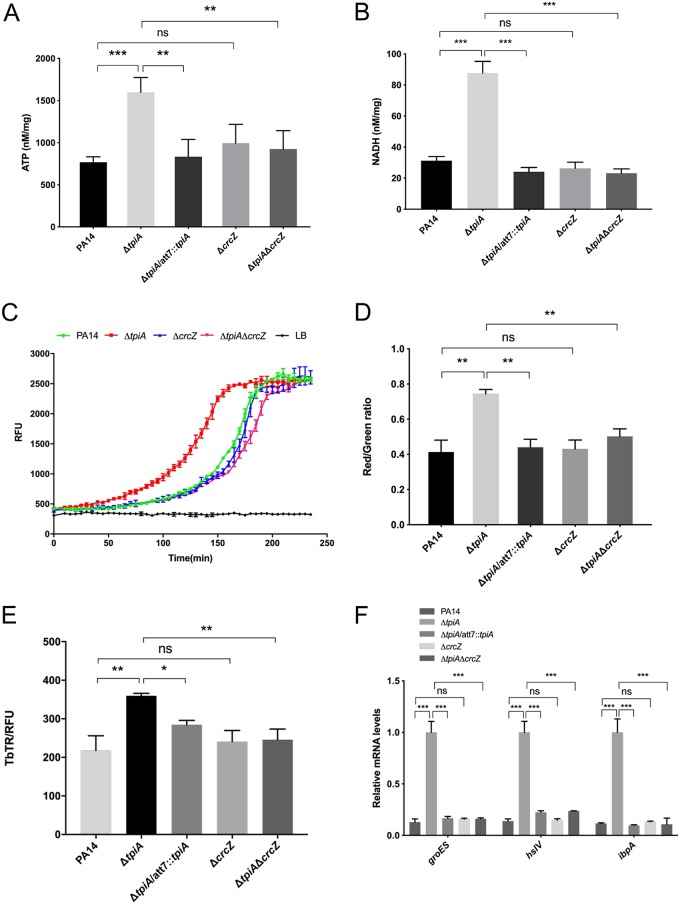
The increased level of CrcZ in the Δ*tpiA* mutant contributes to the hypersusceptibility to tobramycin. (A) The intracellular ATP levels were determined using the enhanced ATP assay kit. Bacteria were cultured in LB medium at 37°C to OD_600_ of 1. A volume of 1.5 ml of the bacteria was collected and lysed by ultrasound sonication. Measurement of the ATP level in the supernatant was performed according to the manufacturer’s instruction. Total protein levels were quantified by BCA analysis for normalization. Data represent the mean ± standard deviation of the results from three samples. **, *P* < 0.01; ***, *P* < 0.001 by Student's *t* test. (B) The intracellular NADH levels were determined using an Amplite fluorimetric NAD/NADH ratio assay kit. Bacteria were cultured in LB medium at 37°C to an OD_600_ of 1. The bacteria were lysed in PBS by sonication. Measurement of the NADH level in the supernatant was performed according to the manufacturer’s instruction. Total proteins were quantified by the BCA analysis for normalization. Data represent the mean ± standard deviation of the results from three samples. **, *P* < 0.01 by Student's *t* test. (C) ETC activity was quantified by an extracellular oxygen consumption assay kit. A total of 1.5 × 10^5^ bacterial cells in fresh LB were mixed with 10 μl of the O_2_ consumption reagent in a black 96-well plate and sealed with 50 μl mineral oil to isolate the air. The fluoresces were measured every 5 min for 4 h. (D) Quantification of PMF-dependent membrane potential by DiOC_2_(3). The logarithmic-phase bacterial cells were incubated with DiOC_2_(3) for 1 h and then subjected to flow cytometry analysis. Data represent the mean ± standard deviation of the results from three samples. **, *P* < 0.01 by Student's *t* test. (E) The internalization of tobramycin was quantified by Texas Red-labeled tobramycin (TbTR). The bacteria were cultured in LB to an OD_600_ of 1, followed by incubation with 40 mg/liter TbTR for 30 min. The bacteria were collected by centrifugation and washed three times with PBS and then lysed in PBS by sonication. The Texas Red was excited at 595 nm, and the emission at 615 nm was measured with a luminometer. The relative fluorescence was normalized by the total protein. Data represent the mean ± standard deviation of the results from three samples. *, *P* < 0.05; **, *P* < 0.01; ns, not significant by Student's *t* test. (F) The bacteria were treated with 0.1 mg/liter tobramycin for 30 min, followed by RNA isolation. The mRNA levels of *groES*, *hslV*, and *ibpA* were determined by real-time PCR. The 30S ribosomal protein gene *rpsL* was used as an internal control. Data represent the mean ± standard deviation of the results from three samples. **, *P* < 0.01 by Student's *t* test.

To examine the role of CrcZ in the Δ*tpiA* mutant, we knocked out *crcZ* in the PA14 and Δ*tpiA* mutant backgrounds. The deletion of *crcZ* in the Δ*tpiA* mutant partially restored the bacterial growth in MHB (Fig. S3B) but fully restored the intracellular ATP and NADH levels, respiratory activity, and membrane potential, as well as the uptake of tobramycin. However, no significant difference was observed between PA14 and its isogenic Δ*crcZ* mutant (Fig. 8A to E). Furthermore, deletion of *crcZ* in the Δ*tpiA* mutant restored the bacterial resistance to aminoglycoside antibiotics but did not affect the resistance in wild-type PA14 (Table 4). When treated with tobramycin, the expression levels of *groES*, *ibpA*, and *hslV* were increased in the Δ*tpiA* mutant but not in the PA14, Δ*tpiA* Δ*crcZ* mutant, and Δ*crcZ* mutant strains (Fig. 8F).

**TABLE 4:** MICs of the aminoglycoside antibiotics against indicated strains in MHB

Strain	MIC (μg/ml)^*a*^				
	GEN	TOB	NEO	STR	AMK
PA14	1	1	16	16	8
Δ*tpiA* mutant	0.25	0.125	4	4	2
Δ*tpiA*/att7::*tpiA* mutant	1	1	16	16	8
Δ*crcZ* mutant	1	1	16	16	8
Δ*tpiA* Δ*crcZ* mutant	1	1	16	16	8

a GEN, gentamicin; TOB, tobramycin; NEO, neomycin; STR, streptomycin; AMK, amikacin.

### CrcZ is involved in repression of the T3SS in the Δ*tpiA* mutant.

**FIG 9:**
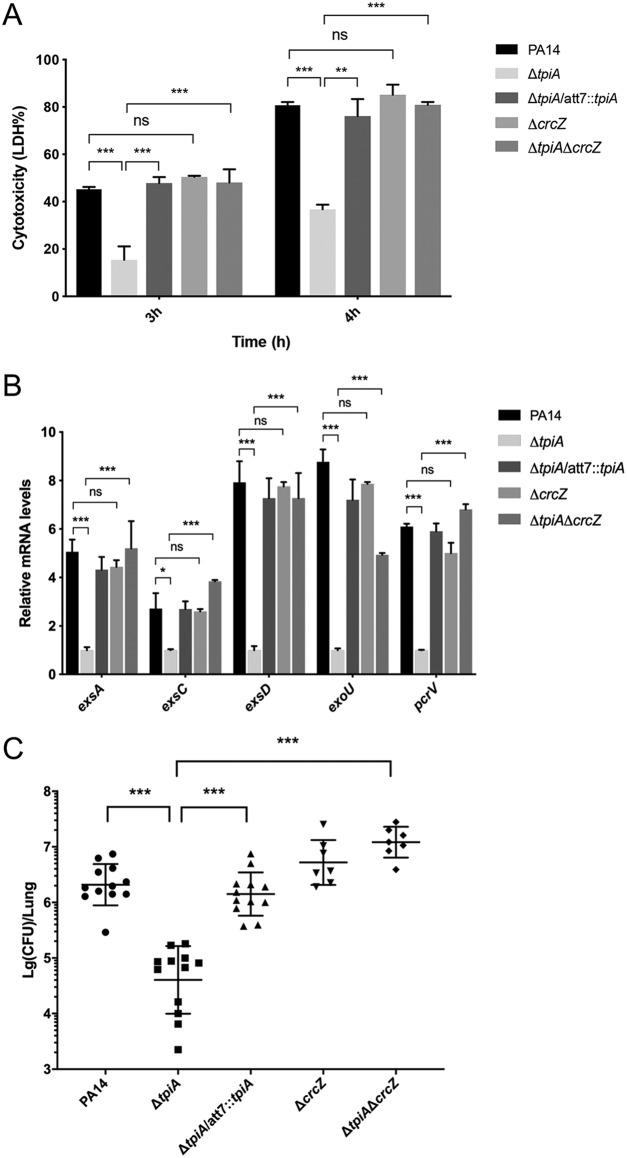
CrcZ represses the T3SS in the Δ*tpiA* mutant. (A) A549 cells were infected with the indicated strains at a multiplicity of infection (MOI) of 50 for 4 h. The relative cytotoxicity was determined by the LDH release assay. Data represent the mean ± standard deviation of the results from three samples. (B) Indicated strains were grown with 5 mM EGTA for 4 h. The relative mRNA levels of the T3SS genes were determined by real-time PCR. The 30S ribosomal protein gene *rpsL* was used as the internal control. Data represent the mean ± standard deviation of the results from three samples. **, *P* < 0.01; ns, not significant by Student's *t* test. (C) Mice were infected intranasally with the indicated strains. At 12 h postinfection, lungs from the infected mice were isolated. Bacterial loads were determined by serial dilution and plating. Each group of 12 mice was infected with the strain of wild-type PA14, Δ*tpiA* mutant, or Δ*tpiA*/att7::*tpiA* mutant. Each group of 7 mice was infected with the Δ*crcZ* mutant or the Δ*tpiA* Δ*crcZ* mutant. ***, *P* < 0.001 by Student's *t* test.

It has been reported that Crc is required for the T3SS in P. aeruginosa (13). We thus hypothesized that the increased level of CrcZ might lead to repression of the T3SS in the Δ*tpiA* mutant. Indeed, expression of the T3SS genes and the cytotoxicity of the Δ*tpiA* mutant were restored by the deletion of *crcZ* (Fig. 9A and B). Since the T3SS plays an important role in acute infections of P. aeruginosa, we examined bacterial virulence in a murine acute pneumonia model. The deletion of *tpiA* attenuated bacterial virulence, which was restored by further deletion of the *crcZ* gene. Meanwhile, the deletion of *crcZ* in PA14 did not affect bacterial virulence (Fig. 9C). In LB, the growth rate of the Δ*tpiA* mutant was lower than that of the wild-type strain but was similar to those of the Δ*crcZ* and Δ*crcZ* Δ*tpiA* mutants. Overexpression of *exsA* in the Δ*tpiA* mutant (Δ*tpiA*/pUCP20-*exsA*) further reduced the growth rate (Fig. S3C). However, the Δ*tpiA*/pUCP20-*exsA* mutant strain displayed the highest cytotoxicity, and the deletion of *crcZ* in the Δ*tpiA* mutant restored bacterial cytotoxicity and virulence in the murine acute pneumonia model. These results suggest that TpiA affects cytotoxicity and bacterial virulence mainly through CrcZ.

Since the *tpiA* mutant shows a growth defect, secondary mutations could occur to compensate the fitness defect. To further confirm that the phenotypes of the mutant strains are related to *tpiA* and *crcZ*, the strains used in this study have been subjected to whole-genome sequencing, including the wild-type PA14 and the three mutant strains, namely, the Δ*tpiA*, Δ*crcZ*, and Δ*tpiA* Δ*crcZ* mutants. In reference to the wild-type strain, the locations of the point mutations are shown in Fig. S4A and listed in Table S3. Frameshift mutations in the CTP synthetase gene *pyrG* were found in all three mutants. A previous study in Mycobacterium tuberculosis demonstrated that a mutation in *pyrG* actually increased bacterial resistance to various antibiotics, including aminoglycoside antibiotics (35). Three mutations were unique in the Δ*tpiA*Δ*crcZ* mutant. The mutated genes are *glgB*, *thiC*, and *PA5393*, encoding a 1,4-α-glucan branching enzyme, a thiamine biosynthesis enzyme, and a hypothetical protein, respectively. Strains with a mutation in each of these genes were obtained from the PA14 transposon insertion mutant library (20). All three mutants displayed the same antibiotic resistance level to tobramycin (MIC, 1 μg/ml) and a similar level of cytotoxicity as those of the wild-type PA14. These results suggest that the decreased antibiotic resistance and cytotoxicity of the *tpiA* mutant are mainly due to the upregulation of CrcZ, ruling out the secondary mutational effect.

10.1128/mBio.02079-19.4 FIG S4 Genomic distribution of detected mutants in deletion isolates. (A) The genomic DNA of the wild-type strain PA14 and the mutant strain was extracted and resequenced, and the mutation position was compared with the PA14 standard genome. The figure indicates the mutation position different from that of the wild-type strain. The circle represents a one-base mutation, and the arrow represents a fragment deletion or fragment insertion mutation. The dotted circle is the common mutation of different strains. The blue arrows represent mutations unique in the Δ*tpiA* Δ*crcZ* mutant. (B) A549 cells were infected with the indicated strains at a multiplicity of infection (MOI) of 50 for 3 h. The relative cytotoxicity was determined using an LDH release assay. Data represent the mean ± standard deviation of the results from three samples. Download FIG S4, PDF file, 0.1 MB. Copyright © 2020 Xia et al. 2020 Xia et al. This content is distributed under the terms of the Creative Commons Attribution 4.0 International license.

10.1128/mBio.02079-19.7 TABLE S3 Mutations in the indicated strains. Download Table S3, XLS file, 0.1 MB. Copyright © 2020 Xia et al. 2020 Xia et al. This content is distributed under the terms of the Creative Commons Attribution 4.0 International license.

## DISCUSSION

Here, we investigated the roles of carbon metabolism-related genes on virulence and aminoglycoside antibiotic susceptibility in P. aeruginosa. We found that mutation of the triosephosphate isomerase gene *tpiA* resulted in the most profound defect in bacterial virulence and antibiotic resistance against aminoglycoside antibiotics.

TpiA is a highly conserved enzyme that catalyzes the interconversion between G3P and DHAP, an important reaction in glycolysis and gluconeogenesis. We suspected that without TpiA, the pyruvate generated from glycolysis cannot be converted into DHAP, thus breaking off the flux of carbon toward the phospholipid synthesis while increasing the TCA cycle. On the other hand, DHAP generated from glycerol cannot be directly converted into G3P in the Δ*tpiA* mutant, which might hinder gluconeogenesis. Overall, these results suggest a critical role of TpiA in carbon metabolism in P. aeruginosa.

Perturbations in metabolism have been found to influence bacterial virulence and resistance to antibiotics. The pyruvate dehydrogenase genes *aceAB* are required for expression of the T3SS genes (36). Histidine and tryptophan utilization have been found to interfere with the T3SS in P. aeruginosa (37, 38). CbrA and CbrB compose a two-component regulatory system that regulates genes involved in carbon and nitrogen utilization. Mutation of *cbrA* results in defective swarming motility, enhanced cytotoxicity and biofilm formation, and increased antibiotic resistance (39). The CbrA/CbrB system directly regulates the expression of the small RNA (sRNA) CrcZ, which antagonizes the Crc/Hfq-mediated CCR (9, 40). Crc is required for the twitching motility, biofilm formation, and expression of the T3SS and quorum sensing system genes, and it contributes to bacterial resistance to various antibiotics (41, 42). Here, we found that the CrcZ level was increased in the Δ*tpiA* mutant. The *cbrA* and *cbrB* mRNA levels and the *crcZ* promoter activity were similar in the Δ*tpiA* mutant and wild-type PA14, indicating a similar level of the CbrA ligand. We further demonstrated that CrcZ stability was increased in the Δ*tpiA* mutant. We suspected that mutation of *tpiA* alters the intracellular metabolite intermediate levels as well as the redox status, which might affect RNase levels and activities (43, 44). Deletion of *crcZ* in the Δ*tpiA* mutant background restored the expression of the T3SS genes and bacterial cytotoxicity, suggesting an important role of CrcZ in repression of the T3SS in the Δ*tpiA* mutant. Recent studies have demonstrated that enhancing bacterial metabolic activity increases the bactericidal effect of antibiotics. For instance, exogenous glutamate stimulates metabolic flux through the pyruvate cycle (P cycle), which increases the aminoglycoside killing efficacy in Escherichia coli and Edwardsiella tarda (32). Supplementation of central carbon metabolites, such as fumarate, succinate, and citrate, increases the susceptibility of P. aeruginosa to tobramycin. The exogenous carbon sources enhance bacterial respiration, thereby increasing membrane potential and the uptake of antibiotics (13, 19, 45–47). Our results revealed that mutation of *tpiA* increased the bacterial respiration and uptake of tobramycin, presumably due to the aberrant carbon flux into the TCA cycle. Therefore, inhibition of TpiA might increase the bacterial killing efficacy of tobramycin and simultaneously decrease bacterial virulence, making TpiA a potential drug target.

## MATERIALS AND METHODS

### Bacterial strains and plasmids.

The bacterial strains, plasmids, and primers used in this study are listed in Table S4. To construct the *tpiA* deletion mutant, a 1,087-bp fragment and a 1,511-bp fragment upstream and downstream of the *tpiA* coding region, respectively, were amplified by PCR using the PA14 chromosome as the template and the primers listed in Table S4. The fragments were cloned into the plasmid pEX18Tc (48). Deletion of the *tpiA* gene in P. aeruginosa was performed as previously described (48). The Δ*crcZ* mutant was constructed by the same method. For the complementation of *tpiA*, the *tpiA* gene and its native promoter were amplified using the primers shown in Table S4. The fragments were ligated into pUC18T-mini-Tn7T-Gm. Integration of the *tpiA* fragment into the bacterial chromosome and removal of the gentamicin resistant cassette were performed as previously described (49).

10.1128/mBio.02079-19.8 TABLE S4 Bacterial strains, plasmids, and primers used in this study. Download Table S4, DOCX file, 0.1 MB. Copyright © 2020 Xia et al. 2020 Xia et al. This content is distributed under the terms of the Creative Commons Attribution 4.0 International license.

Bacteria were cultured in LB medium at 37°C. Antibiotics were used at the following concentrations: for E. coli, 10 μg ml^−1^ tetracycline, 100 μg ml^−1^ ampicillin, and 10 μg ml^−1^ gentamicin; and for P. aeruginosa, 50 μg ml^−1^ gentamicin, 50 μg ml^−1^ tetracycline, and 150 μg ml^−1^ carbenicillin.

The basic artificial sputum medium (ASM) is composed of 5 g mucin from pig stomach mucosa (NBS Biologicals), 5.9 mg diethylenetriaminepentaacetic acid (DTPA) (Sigma), 4 g low-molecular-weight salmon sperm DNA (Fluka), 1.81 g Tris base (Sigma), 2.2 g KCl, 5 g NaCl, 5 ml egg yolk emulsion (Oxoid), and 5 g Casamino Acids (Difco) per 1 liter of water (pH 7.0).

The maximum growth rate (MGR) was calculated as (W7 − W4)/3, where W7 and W4 represent the bacterial OD_600_ at 7 h and 4 h, respectively.

### Antibiotic susceptibility test.

The MIC was determined using broth microdilution in accordance with Clinical and Laboratory Standards Institute (CLSI) recommendations (http://iacld.ir/DL/public/CLSI-2018-M100-S28.pdf). Each well of the 96-well microtiter plate was filled with 0.1 ml Mueller-Hinton broth (MHB) with serially diluted concentrations of the individual antibiotics. Then, 0.1 ml bacterial culture (5 × 10^5^ CFU) was inoculated into each well and incubated at 37°C for 20 h. MICs were recorded as the lowest concentration of antibiotic inhibiting visible growth. The same procedure was used to determine the MICs in ASM. All of the MICs of the strains were tested at least three times.

### Transcriptome sequencing and analysis.

Bacteria were cultured in LB medium at 37°C until late-logarithmic phase (OD_600_, 1). Total RNA was isolated using the RNAprep pure cell/bacteria kit (Tiangen Biotec, Beijing, China). Two replicates were prepared for each strain. Sequencing and analysis services were performed as previously described (50). The detailed method is described in Text S1 in the supplemental material.

10.1128/mBio.02079-19.9 TEXT S1 Transcriptome sequencing and analysis and DNA resequencing methods. Download Text S1, DOCX file, 0.1 MB. Copyright © 2020 Xia et al. 2020 Xia et al. This content is distributed under the terms of the Creative Commons Attribution 4.0 International license.

### Real-time PCR.

Total bacterial RNA was isolated using the RNAprep pure cell/bacteria kit (Tiangen Biotec, Beijing, China). cDNAs were synthesized with a reverse transcriptase and random primers (TaKaRa, Dalian, China). Real-time PCR was performed using the SYBR II green supermix (Bio-Rad, Beijing, China). The 30S ribosomal protein gene *rpsL* was used as the internal control.

### Measurement of the intracellular DHAP level.

Bacterial intracellular DHAP levels were determined using a high-sensitivity dihydroxyacetone phosphate assay kit (catalog no. MAK275; Sigma-Aldrich, USA). Bacteria were cultured in M9 medium with 20 mM glucose and 40 mM glycerol as the carbon sources at 37°C to an OD_600_ of 1. Then, the bacteria were washed three times with phosphate-buffered saline (PBS) and incubated in M9 medium with 40 mM glucose or 80 mM glycerol as the sole carbon source for 1 h. The bacterial cells were lysed in the PBS buffer by sonication. Measurement of the DHAP level in the supernatant was performed according to the manufacturer’s instructions. The standard curve was prepared using a standard DHAP provided by the kit. The total protein level was quantified using a bicinchoninic acid (BCA) analysis kit (Beyotime, Shanghai, China).

### Tobramycin-Texas Red uptake assay.

Tobramycin was conjugated to Texas Red as previously described (19, 51). Briefly, 1 μg Texas Red sulfonyl chloride was dissolved in 50 μl anhydrous *N,N*-dimethyl formamide on ice. Then, the solution was added slowly to 2.3 ml of 0.1 M K_2_CO_3_ (pH 8.5) with or without 10 mg/ml tobramycin. To quantify the uptake of the tobramycin-Texas Red (TbTR), bacteria were grown in LB to an OD_600_ of 1 and then incubated with 40 mg/liter TbTR or Texas Red for 30 min. The bacteria were collected by centrifugation and washed three times with PBS. The bacterial cells were lysed in PBS by sonication. The Texas Red was excited at 595 nm, and the emission at 615 nm (19, 51) was measured with a luminometer (Varioskan Flash; Thermo Scientiﬁc). Total bacterial protein concentrations were quantified using a BCA analysis for calibration.

### Bacterial ATP level assay.

The intracellular ATP levels were determined using an enhanced ATP assay kit (Beyotime Biotec, Shanghai, China). Bacteria were cultured in LB medium at 37°C until late-logarithmic phase (OD_600_, 1). Bacterial cells in 1.5 ml culture were collected by centrifugation and resuspended using the lysis buffer provided by the kit. The cells were broken by ultrasound sonication. Then, the cell debris was removed by centrifugation. One hundred microliters of the ATP detection working fluid was mixed with 20 μl of the bacterial supernatant. Chemiluminescence was detected with a luminometer (Varioskan Flash; Thermo Scientiﬁc). Total proteins were quantified by the BCA analysis for calibration. Standard curves were prepared using the standard ATP samples provided by the kit.

### Bacterial NADH level assay.

Bacterial NADH levels were determined using an Amplite fluorimetric NAD/NADH ratio assay kit (AAT Bioquest, Inc., USA). Bacteria were cultured in LB medium at 37°C to an OD_600_ of 1. The bacterial cells were lysed in PBS by sonication. Measurement of the NADH level in the supernatant was performed according to the manufacturer’s instructions. The standard curve was prepared using the NADH provided by the kit. The total protein level was quantified using a BCA analysis kit (Beyotime, Shanghai, China).

### Membrane potential assay.

The bacteria were cultured at 37°C in LB medium to an OD_600_ of 1. The bacteria were diluted to 10^6^ CFU/ml in PBS. To each 1.5 ml of the bacterial suspension, 2 μl of 20 mM DiOC_2_(3) was added and incubated for 1 h at 37°C in dark, followed by ﬂow cytometry analysis (Accuri C6; BD). The DiOC_2_(3) was excited at 484 nm, and the emissions at 530 nm (green ﬂuorescence) and 610 nm (red ﬂuorescence) were measured. Cells without staining were subjected to the fluorescence measurement, and the values were subtracted from those of the corresponding cells with DiOC_2_(3) staining. All experiments were performed at least in triplicate. The green fluorescence is relatively independent of membrane potential and mainly reflects particle sizes, while the red fluorescence is highly dependent on membrane potential and particle sizes. Membrane potential was indicated by the ratio of the green to red ﬂuorescence (30).

### Oxygen consumption assay.

Bacterial oxygen consumption was determined using an extracellular oxygen consumption assay kit (Abcam), according to the manufacturer’s instructions. Brieﬂy, overnight bacterial cultures were diluted 1:100 in fresh LB medium and grown at 37°C until the OD_600_ reached 1. The bacterial concentration was adjusted to 1 × 10^6^ CFU/ml in fresh LB medium. One hundred fifty microliters of the bacterial resuspension was transferred to each well of a 96-well plate that contained 10 μl of O_2_ consumption reagent. Each well was sealed with 50 μl mineral oil provided by the kit to isolate the air. The dye excites at 380 nm and emits at 650 nm, and the signal was monitored with a luminometer (Varioskan Flash; Thermo Scientiﬁc).

### β-Galactosidase assay.

Overnight bacterial cultures were diluted 1:100 in fresh LB and grown at 37°C until the OD_600_ reached 1. Bacterial cells in 0.5 ml culture were collected and resuspended in 1.5 ml Z buffer (60 mM Na_2_HPO_4_, 60 mM NaH_2_PO_4_, 1 mM MgSO_4_, 10 mM KCl, 50 mM β-mercaptoethanol [pH 7.0]). The β-galactosidase activity was determined as previously described (52).

### RNA stability analysis.

P. aeruginosa strains were grown in LB at 37°C to an OD_600_ of 1. Then, the bacteria were treated with 100 μg/ml rifampin to stop transcription. At each indicated time point, the bacterial cells were taken and mixed with an equal number of E. coli cells expressing the *gfp* gene. Total RNA was puriﬁed, and the levels of *crcZ* were analyzed by real-time PCR. The *gfp* RNA level was used as an internal control for normalization.

### Cytotoxicity assay.

Cytotoxicity was determined using a lactate dehydrogenase (LDH) release assay. The human lung carcinoma cell line A549 cells were cultured in Dulbecco’s modiﬁed Eagle medium with 2% (vol/vol) heat-inactivated fetal bovine serum at 37°C with 5% CO_2_. A total of 2 × 10^5^ cells were seeded into each well of a 24-well plate and cultured overnight. The bacterial cells were cultured at 37°C in LB medium to an OD_600_ of 1 and then washed twice with PBS and resuspended in PBS. After the addition of the bacteria to each well, the plate was centrifuged at 700 × *g* for 10 min to synchronize the infection. Four hours postinfection, LDH in the medium was measured using the LDH cytotoxicity assay kit (Beyotime, Shanghai, China). Cells treated with the LDH release buﬀer provided by the kit were used as the control for the total LDH. The cytotoxicity percentage was calculated according to the manufacturer’s instructions.

### Murine acute pneumonia model.

The animal infection experiments were performed following the National and Nankai University guidelines on the use of animals in research. The protocol (permit number NK-04-2012) was approved by the animal care and use committee of the College of Life Sciences of Nankai University. The infection of mice was performed as previously described (53). Brieﬂy, overnight bacterial culture was diluted 1:100 in fresh LB and grown at 37°C until the OD_600_ reached 1. The collected bacterial cells were washed once with PBS. The bacterial concentration was adjusted to 2 × 10^8^ CFU/ml in PBS. Each female BALB/c mouse (Vital River, Beijing, China) at the age of 6 to 8 weeks was anesthetized with an intraperitoneal injection of 100 μl of 7.5% chloral hydrate, followed by inoculation with 20 μl of the bacterial suspension, resulting in 4 × 10^6^ CFU per mouse. Twelve hours postinfection, the mice were sacriﬁced. Lungs were isolated and subjected to homogenization. The bacterial load in each lung was determined by plating.
