# Supplementary material for: TpiA is a Key Metabolic Enzyme That Affects Virulence and Resistance to Aminoglycoside Antibiotics through CrcZ in Pseudomonas aeruginosa
Source: mBio. 2020 Jan 7;11(1):e02079-19. doi: 10.1128/mBio.02079-19 (PMC6946797; doi:10.1128/mBio.02079-19)
Supplement: FIG S1 [file mBio.02079-19-sf001.pdf]

Fig.S1

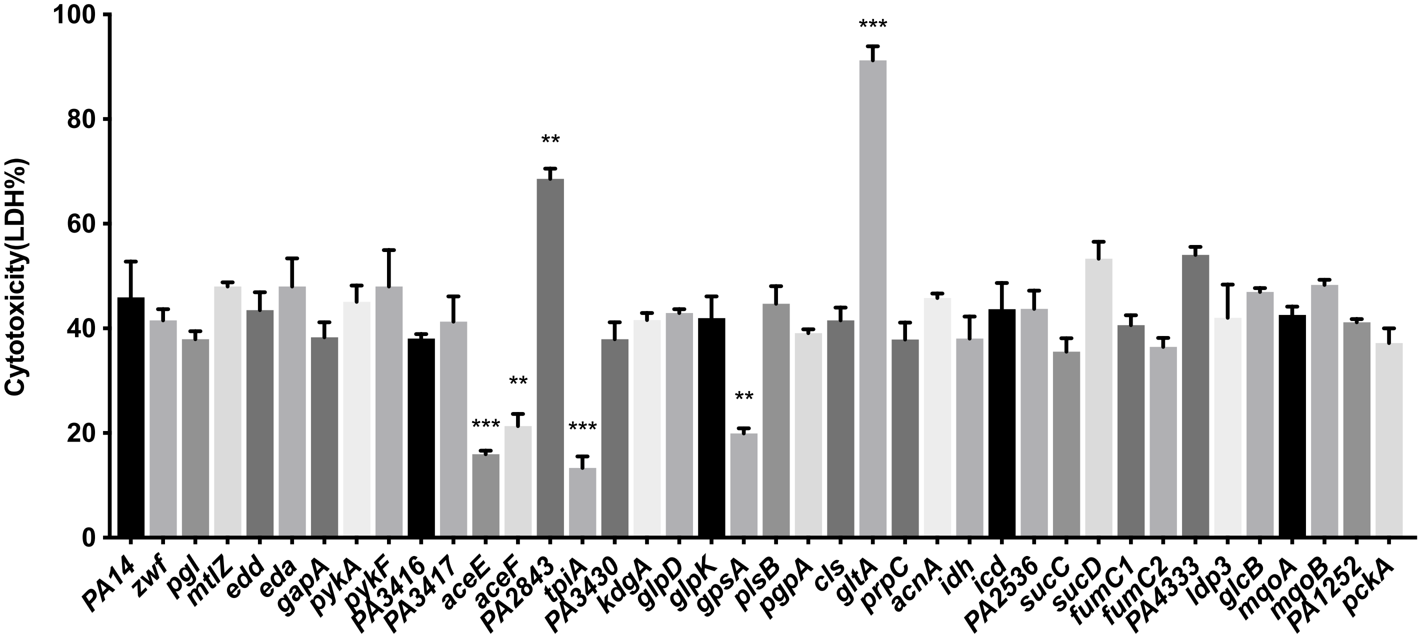

Fig. S1. Cytotoxicity of strains with mutation in carbon metabolism related genes. A549 cells were infected with indicated strains at an MOI of 50 for 3 h. The relative cytotoxicity was determined by the LDH release assay. **, P < 0.01; ***, P < 0.001 compared to PA14 by Student's t-test.
