# Supplementary material for: TpiA is a Key Metabolic Enzyme That Affects Virulence and Resistance to Aminoglycoside Antibiotics through CrcZ in Pseudomonas aeruginosa
Source: mBio. 2020 Jan 7;11(1):e02079-19. doi: 10.1128/mBio.02079-19 (PMC6946797; doi:10.1128/mBio.02079-19)
Supplement: TABLE S1 [file mBio.02079-19-st001.docx]

**Table S1. Bacterial resistance levels to tobramycin in Mueller Hinton broth.** The altered MICs were highlighted in bold.

| Gene with Tn insertion | Wild type | *zwf* | *pgl* | *mtlZ* | *edd* | *eda* | *gapA* | *pykA* | *pykF* | *PA3416* |
| --- | --- | --- | --- | --- | --- | --- | --- | --- | --- | --- |
| MIC (μg/ml) | 1 | **0.5** | 1 | 1 | 1 | 1 | 1 | 1 | **2** | 1 |
| Gene with Tn insertion | *tpiA* | PA3430 | *kdgA* | *glpD* | *glpK* | *gpsA* | *plsB* | *pgpA* | *cls* | *gltA* |
| MIC (μg/ml) | **0. 25** | 1 | 1 | 1 | 1 | 1 | 1 | 1 | 1 | **2** |
| Gene with Tn insertion | *sucC* | *sucD* | *fumC1* | *fumC2* | PA4333 | *aceA* | *glcB* | *mqoA* | *mqoB* | PA1252 |
| MIC (μg/ml) | 1 | 1 | **2** | 1 | 1 | 1 | 1 | 1 | 1 | 1 |
| Gene with Tn insertion | PA3417 | *Lpd3* | *aceF* | PA2843 | *prpC* | *acnA* | *idh* | *icd* | *pckA* | PA2536 |
| MIC (μg/ml) | 1 | **2** | 1 | 1 | 1 | 1 | 1 | 1 | 1 | 1 |
