# Supplementary material for: TpiA is a Key Metabolic Enzyme That Affects Virulence and Resistance to Aminoglycoside Antibiotics through CrcZ in Pseudomonas aeruginosa
Source: mBio. 2020 Jan 7;11(1):e02079-19. doi: 10.1128/mBio.02079-19 (PMC6946797; doi:10.1128/mBio.02079-19)
Supplement: FIG S2 [file mBio.02079-19-sf002.pdf]

Fig.S2

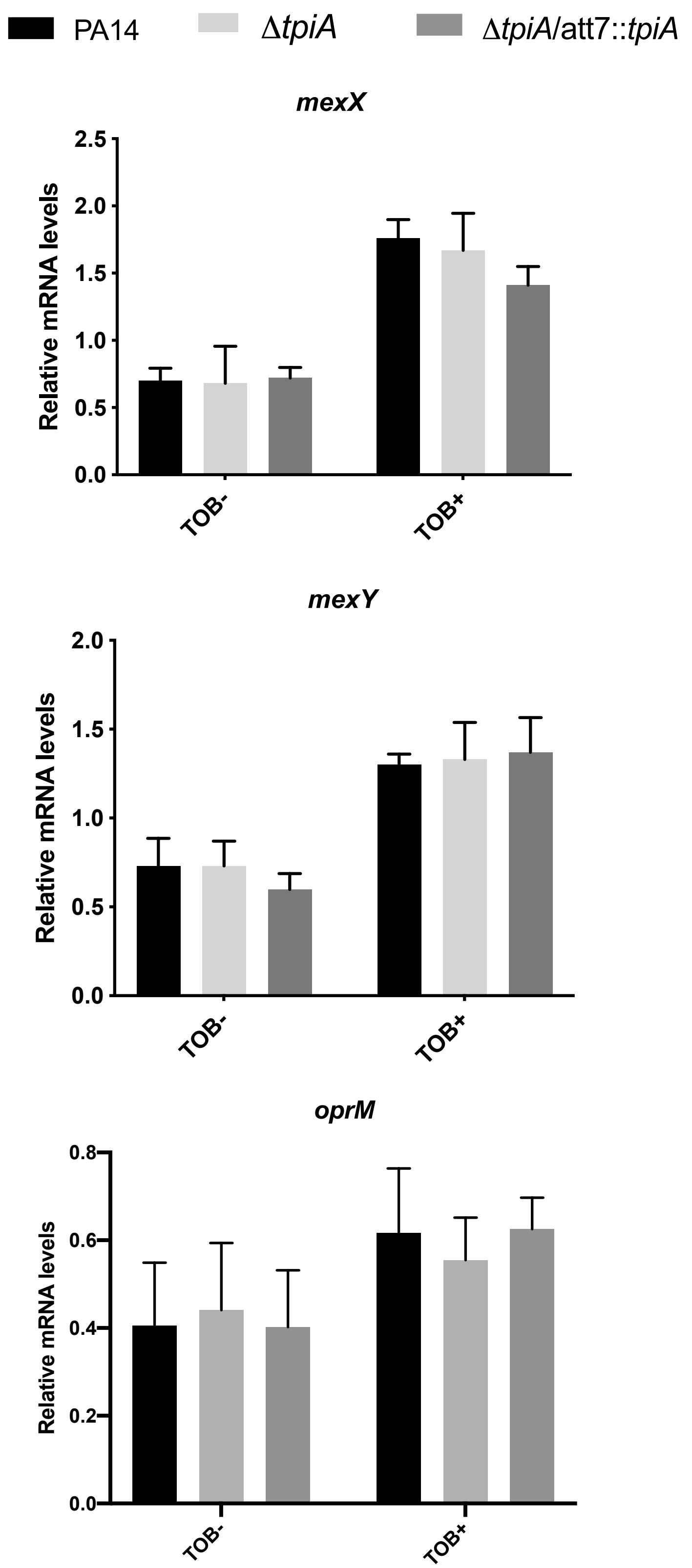

Fig. S2. Expression levels of the efflux pump genes in the Δ*tpiA* mutant. The bacteria were cultured in LB to an OD_{600} of 1, and then incubated with or without 0.1 mg/L tobramycin for 30 min. The mRNA levels of indicated genes were determined by real time PCR. The 30S ribosomal protein gene *rpsL* was used as an internal control. Results represent means ± SD.
