# Supplementary material for: TpiA is a Key Metabolic Enzyme That Affects Virulence and Resistance to Aminoglycoside Antibiotics through CrcZ in Pseudomonas aeruginosa
Source: mBio. 2020 Jan 7;11(1):e02079-19. doi: 10.1128/mBio.02079-19 (PMC6946797; doi:10.1128/mBio.02079-19)
Supplement: FIG S3 [file mBio.02079-19-sf003.pdf]

Fig.S3

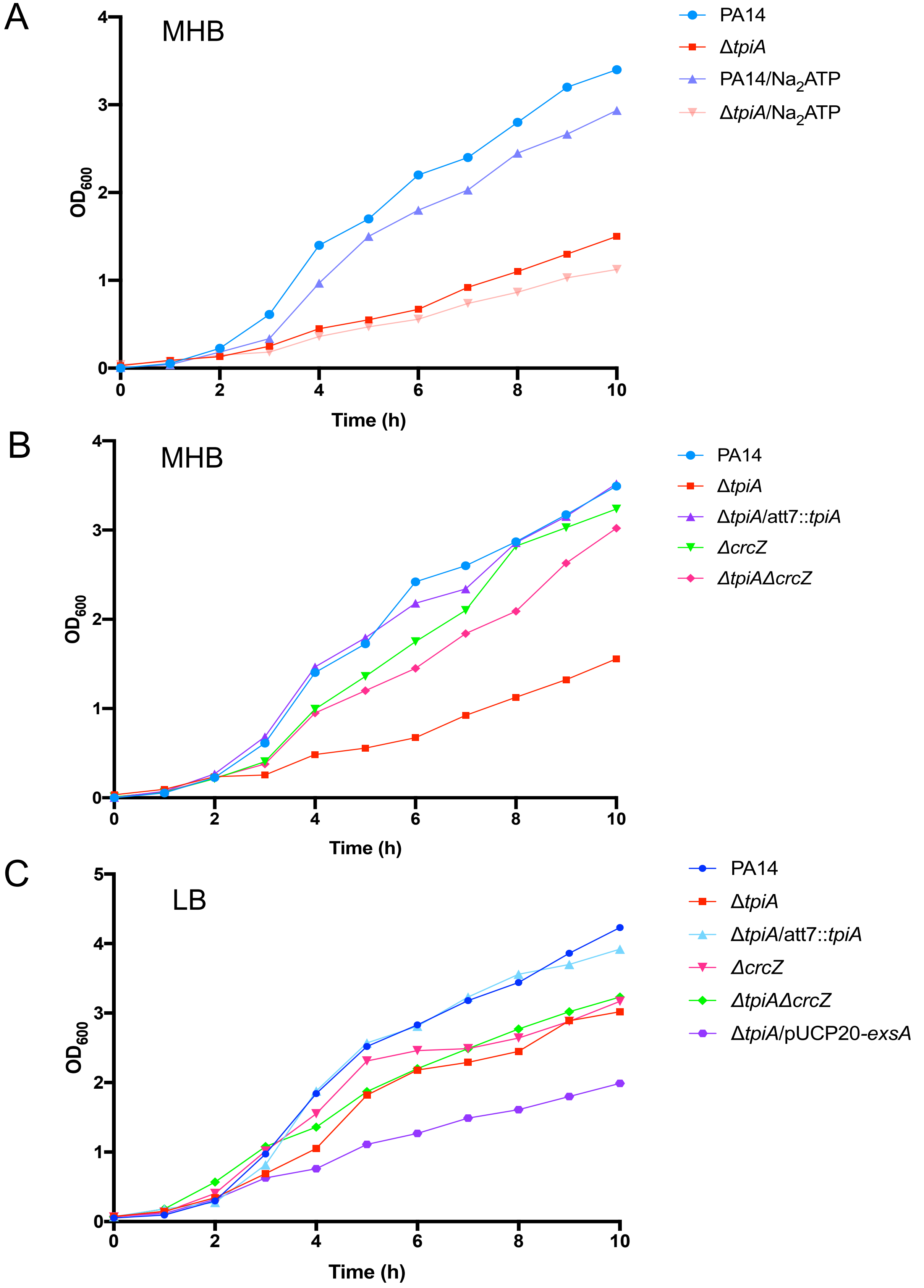

Fig. S3. Growth curves of bacteria in different media. Overnight cultures of indicated strains were 1:100 diluted into fresh LB. The bacterial growth was monitored by measuring OD_{600} every hour for 10 hours. (A) The growth curves of indicated strains grown in MHB with or without 5mM Na_2ATP. The growth curves of indicated strains grown in MHB (B) and LB (C).
