# Supplementary material for: TpiA is a Key Metabolic Enzyme That Affects Virulence and Resistance to Aminoglycoside Antibiotics through CrcZ in Pseudomonas aeruginosa
Source: mBio. 2020 Jan 7;11(1):e02079-19. doi: 10.1128/mBio.02079-19 (PMC6946797; doi:10.1128/mBio.02079-19)
Supplement: TABLE S2 [file mBio.02079-19-st002.docx]

**Table S2. Expression levels of carbon metabolism related genes.** Genes with similar expression patterns in a *hfq* (1) and a *crc* mutant (2) were shown in red and blue, respectively.

| Symbol | PAO1 | | PA14 | Product | Fold change Δ*tpiA*/PA14 | | P-value | |
| --- | --- | --- | --- | --- | --- | --- | --- | --- |
| Glycolysis |  | |  |  | |  |  | |
| *glpK* | PA3582 | | PA14_17960 | glycerol kinase; | | 2.0048 | 0 | |
| *zwf* | PA3183 | | PA14_23070 | glucose-6-phosphate 1-dehydrogenase; | | 0.3702 | 0 | |
| Oxidative phosphorylation | | | |  | |  |  | |
| *coxA* | PA0106 | | PA14_01300 | cytochrome c oxidase subunit I; | | 5.0759 | 0 | |
| *coxB* | PA0105 | | PA14_01290 | cytochrome c oxidase subunit II; | | 6.8636 | 0 | |
| *coIII* | PA0108 | | PA14_01320 | cytochrome c oxidase subunit III; | | 4.6898 | 0 | |
| NA | PA4133 | | PA14_10500 | cbb3-type cytochrome c oxidase subunit I; | | 2.0161 | 1.89E-101 | |
| *cioA* | PA3930 | | PA14_13030 | CioA, cyasznide insensitive terminal oxidase; | | 3.1415 | 0 | |
| NA | PA0107 | | PA14_01310 | cytochrome C oxidase assembly protein; | | 7.1372 | 3.56E-103 | |
| NA | PA0112 | | PA14_01360 | putative cytochrome oxidase assembly protein | | 5.5050 | 1.33E-97 | |
| Pyruvate metabolism | | |  |  | |  |  | |
| NA | PA3417 | | PA14_19900 | pyruvate dehydrogenase E1 component subunit alpha; | | 12.4656 | 1.02E-248 | |
| NA | PA3416 | | PA14_19910 | pyruvate dehydrogenase E1 component, beta chain; | | 8.6532 | 1.52E-90 | |
| NA | PA3415 | | PA14_19920 | branched-chain alpha-keto acid dehydrogenase subunit E2; | | 3.4649 | 2.11E-41 | |
| *lpdV* | PA2250 | | PA14_35490 | dihydrolipoamide dehydrogenase; | | 2.3682 | 0 | |
| *bauA* | PA0132 | | PA14_01620 | beta alanine--pyruvate transaminase; | | 2.4271 | 0 | |
| *lldA* | PA2382 | | PA14_33860 | L-lactate dehydrogenase; | | 2.1766 | 2.21E-41 | |
| TCA cycle | |  |  |  | |  |  | |
| *acnA* | PA1562 | | PA14_44290 | aconitate hydratase; | | 3.8276 | 0 | |
| *fumC2* | PA0854 | | PA14_53220 | fumarate hydratase; | | 2.0189 | 7.18E-144 | |
| *lpdV* | PA2250 | | PA14_35490 | dihydrolipoamide dehydrogenase; | | 2.3682 | 0 | |
| *mqoA* | PA3452 | | PA14_19470 | malate:quinone oxidoreductase; | | 0.2443 | 0 | |
| *pckA* | PA5192 | | PA14_68580 | phosphoenolpyruvate carboxykinase; | | 0.4185 | 0 | |
| Lipid β-oxidation | | |  |  | |  | |  |
| *fadE* | PA2815 | | PA14_27730 | acyl-CoA dehydrogenase; | | 3.7958 | 0 | |
| NA | PA3454 | | PA14_19430 | acyl-CoA thiolase; | | 2.0091 | 8.67E-33 | |
| NA | PA4785 | | PA14_63250 | acetyl-CoA acetyltransferase; | | 2.0847 | 5.20E-22 | |
| *fadJ* | PA1737 | | PA14_42080 | 3-hydroxyacyl-CoA dehydrogenase; | | 2.1933 | 2.93E-79 | |
| *faoA* | PA3014 | | PA14_25080 | multifunctional fatty acid oxidation complex subunit alpha; | | 2.1471 | 0 | |
| NA | PA3426 | | PA14_19740 | enoyl-CoA hydratase; | | 2.3173 | 2.30E-67 | |
| *fabH2* | PA3333 | | PA14_20950 | 3-oxoacyl-ACP synthase; | | 9.4472 | 0 | |
| NA | PA1470 | | PA14_45430 | short chain dehydrogenase; | | 2.2092 | 2.08E-39 | |
| *fabG* | PA4786 | | PA14_63270 | 3-ketoacyl-ACP reductase; | | 2.4342 | 5.54E-179 | |
| NA | PA4435 | | PA14_57610 | probable acyl-CoA dehydrogenase | | 3.4946 | 0 | |
| *amiR* | PA3363 | | PA14_20590 | aliphatic amidase regulator; | | 2.5786 | 4.34E-71 | |
| *amiC* | PA3364 | | PA14_20580 | aliphatic amidase expression-regulating protein; | | 2.5153 | 4.35E-147 | |
| NA | PA2553 | | PA14_31530 | acyl-CoA thiolase; | | 3.1448 | 0 | |
| *amiE* | PA3366 | | PA14_20560 | acylamide amidohydrolase; | | 2.4081 | 0 | |
| Lipid biosynthesis | | |  |  | |  |  | |
| *accC* | PA4848 | | PA14_64110 | biotin carboxylase | | 0.4903 | 0 | |
| NA | PA0494 | | PA14_06450 | acetyl-CoA carboxylase biotin carboxylase subunit; | | 0.1321 | 0 | |
| *pfm* | PA2950 | | PA14_25900 | trans-2-enoyl-CoA reductase; | | 0.3507 | 0 | |
| *fabD* | PA2968 | | PA14_25650 | malonyl-CoA-ACP transacylase; | | 0.3987 | 0 | |
| Amino acid metabolism | | | |  | |  |  | |
| *mmsA* | PA3570 | | PA14_18120 | methylmalonate-semialdehyde dehydrogenase; | | 2.5592 | 8.67E-112 | |
| *bauA* | PA0132 | | PA14_01620 | beta alanine--pyruvate transaminase; | | 5.3906 | 0 | |
| NA | PA4435 | | PA14_57610 | probable acyl-CoA dehydrogenase | | 3.4946 | 0 | |
| *pauA3* | PA1566 | | PA14_44240 | glutamine synthetase; | | 7.9081 | 1.11E-304 | |
| *ldh* | PA3418 | | PA14_19870 | leucine dehydrogenase; | | 7.9154 | 0 | |
| *mmsB* | PA3569 | | PA14_18140 | 3-hydroxyisobutyrate dehydrogenase; | | 4.4349 | 2.96E-66 | |
| NA | PA4128 | | PA14_10570 | 2,4-dihydroxyhept-2-ene-1,7-dioic acid aldolase; | | 4.2482 | 3.50E-247 | |
| *bkdA1* | PA2247 | | PA14_35530 | 2-oxoisovalerate dehydrogenase subunit alpha; | | 2.4089 | 0 | |
| *bkdA2* | PA2248 | | PA14_35520 | 2-oxoisovalerate dehydrogenase subunit beta; | | 2.8397 | 0 | |
| *lpdV* | PA2250 | | PA14_35490 | dihydrolipoamide dehydrogenase; | | 2.3682 | 0 | |
| *bkdB* | PA2249 | | PA14_35500 | branched-chain alpha-keto acid dehydrogenase subunit E2; | | 2.7170 | 0 | |
| *liuE* | PA2011 | | PA14_38490 | hydroxymethylglutaryl-CoA lyase; | | 2.2267 | 0 | |
| *kynU* | PA2080 | | PA14_37610 | kynureninase; | | 2.0448 | 4.30E-105 | |
| NA | PA2079 | | PA14_37630 | amino acid permease; | | 2.5942 | 2.70E-141 | |

**References**

1. Sonnleitner E, Wulf A, Campagne S, Pei XY, Wolfinger MT, Forlani G, Prindl K, Abdou L, Resch A, Allain FH, Luisi BF, Urlaub H, Blasi U. 2018. Interplay between the catabolite repression control protein Crc, Hfq and RNA in Hfq-dependent translational regulation in *Pseudomonas aeruginosa*. Nucleic Acids Res 46:1470-1485.

2. Corona F, Martinez JL, Nikel PI. 2019. The global regulator Crc orchestrates the metabolic robustness underlying oxidative stress resistance in *Pseudomonas aeruginosa*. Environ Microbiol 21:898-912.
