# Supplementary material for: TpiA is a Key Metabolic Enzyme That Affects Virulence and Resistance to Aminoglycoside Antibiotics through CrcZ in Pseudomonas aeruginosa
Source: mBio. 2020 Jan 7;11(1):e02079-19. doi: 10.1128/mBio.02079-19 (PMC6946797; doi:10.1128/mBio.02079-19)
Supplement: FIG S4 [file mBio.02079-19-sf004.pdf]

Fig.S4

A

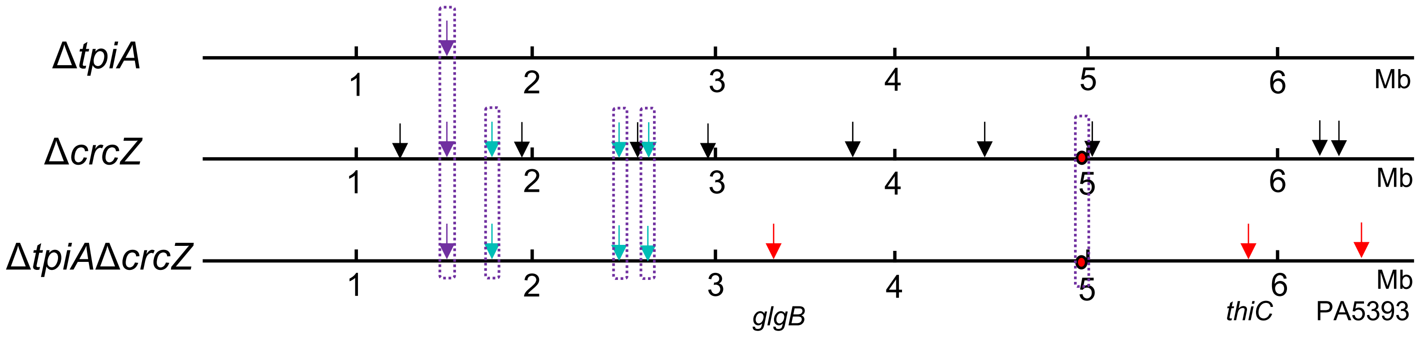

B

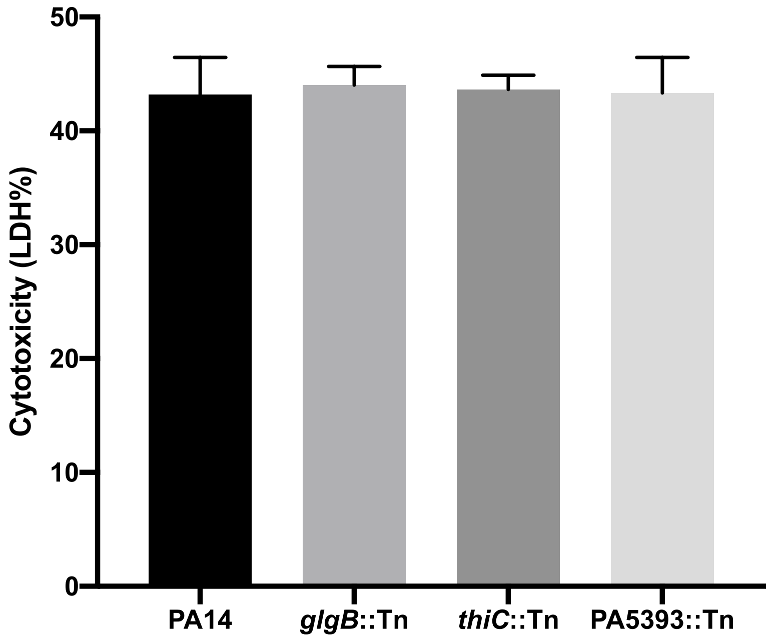

Fig. S4. Genomic distribution of detected mutants in deletion isolates. (A) The genomic DNA of the wild-type strain PA14 and the mutant strain was extracted and resequenced, and the mutation position was compared with the PA14 standard genome. The figure indicates the mutation position different from that of the wild-type strain. The circle represents a base mutation with one, and the arrow represents a fragment deletion or fragment insertion mutation. The dotted circle is the common mutation of different strains. The blue arrows represent mutations unique in *ΔtpiAΔcrcZ.* (B) A549 cells were infected with the indicated strains at an MOI of 50 for 3 h. The relative cytotoxicity was determined by the LDH release assay.
