# Supplementary material for: TpiA is a Key Metabolic Enzyme That Affects Virulence and Resistance to Aminoglycoside Antibiotics through CrcZ in Pseudomonas aeruginosa
Source: mBio. 2020 Jan 7;11(1):e02079-19. doi: 10.1128/mBio.02079-19 (PMC6946797; doi:10.1128/mBio.02079-19)
Supplement: TABLE S4 [file mBio.02079-19-st004.docx]

**Table S4. Bacterial strains, plasmids and primers used in this study.** The enzymes sites are indicated by the underline

| **Strain/ Plasmid /Primer** | **Description** | | **Source (Reference)** |
| --- | --- | --- | --- |
| ***P. aeruginosa*** | | | |
| PA14 | | Wild type strain of *Pseudomonas aeruginosa* | (1) |
| △*tpiA* | | PA14 deleted of *tpiA* | This study |
| △*tpiA*/att7::*tpiA* | | PA14 deleted of *tpiA* with *tpiA* inserted on chromosome with mini-Tn7T insertion; GEN^r^ | This study |
| △*tpiA*/pUCP20-*exsA* | | PA14 deleted of *tpiA* with plasmid pUCP20-*exsA*; CAR^r^ | This study |
| △*crcZ* | | PA14 deleted of *crcZ* | This study |
| △*tpiA*△*crcZ* | | PA14 deleted of *tpiA* and *crcZ* | This study |
| **Plasmid** | |  |  |
| pUCP20 | | *Escherichia–Pseudomonas* shuttle vector without lac promoter; AMP^r^ | (2) |
| pEX18Tc | | Gene replacement vector; TET ^r^, *oriT*^+^, *sacB*^+^ | (2) |
| pMMB67EH | | Expression vector with *tac* promoter; AMP^r^ | (3) |
| pUC18T-mini-Tn7T-Gm  pFLP2  pRKaraRed | | mini-Tn7 base vector from insertion into chromosome attTn7 site; GEN^r^  site-specific excision of GEN^r^ cassette; AMP^r^  Expression vector with P*_BAD_* promoter; TET^r^ | (2)  (4)  (5) |
| pUC18T-mini-Tn7T-Gm-*tpiA* | | pUC18T-mini-Tn7T-Gm with *tpiA*; GEN^r^ | This study |
| pEX18Tc-△*tpiA* | | *tpiA* gene of PA14 deletion on pEX18Tc; TET^r^ | This study |
| pEX18Tc-△*crcZ* | | *crcZ* gene of PA14 deletion on pEX18Tc; TET^r^ | This study |
| pMMB67EH-*amiE**-LacZ | | *amiE* CRC regulatory sequence of PA14 fused to LacZ on pMMB67EH; AMP^r^ | This study |
| pRKaraRed-*amiE*-His | | *amiE* -His on pRKaraRed; TET^r^ | This study |
| pUCP20-*exsA* | | Overpression of *exsA* on pUCP20; CAR^r^ | This study |
| **Primer** | | **Sequence (5’→3’)** | **Function** |
| TpiA-L-F | | CCCGAGCTCCCGATGACCGTGGTGGAGT | *tpiA* deletion |
| TpiA-L-R | | TGCTCTAGAAGCTGACGCAAGCCTTTGA | *tpiA* deletion |
| TpiA-R-F | | TGCTCTAGAGAGCCTCCCTCAATGCGG | *tpiA* deletion |
| TpiA-R-R | | CCCAAGCTTGTAGCTGCCGTTATGACGATTG | *tpiA* deletion |
| TpiA-Pro-L | | CCCGAGCTCATCGGCGATCATCCGGTAG | *tpiA* cloning |
| TpiA-Pro-R | | TACTCAGGATCCTCAATTGACTACCCTCTGAAGCC | *tpiA* cloning |
| TpiA-L | | TACTCAGAATTCTTCTGAGGTATGTGCTTGATTTTG | *tpiA* cloning |
| TpiA-R | | TACTCAGGATCCCCAACAAGACCCAATGCCA | *tpiA* cloning |
| CrcZ-L-F | | CCGGAATTCTGTTCCTCGACGAGATCGG | *crcZ* deletion |
| CrcZ-L-R | | CGCGGATCCTGTTGTTGTGCCAATACATAAGC | *crcZ* deletion |
| CrcZ-R-F | | CGGGGTACCGCTGGACGAGATGGCGCG | *crcZ* deletion |
| CrcZ-R-R | | CCCAAGCTTCAGTTCGTACTCGACCAGCAGT | *crcZ* deletion |
| AmiE*-F | | CCGGAATTCGGGACCGAACCTAACGCATA | *amiE**-lacZ cloning |
| AmiE*-R | | CGCGGATCCGCTGGAAATATCGCCGTGAC | *amiE**-lacZ cloning |
| LacZ-F | | CGCGGATCCGTTTTACAACGTCGTGACTGG | *amiE**-lacZ cloning |
| LacZ-R | | CCCAAGCTTTTTCCTTACGCGAAATACGG | *amiE**-lacZ cloning |
| AmiE-His-F | | CCGGAATTCGCGCATCAGCGTCGATGT | *amiE-*His cloning |
| AmiE-His-R | | CCAAGCTTTCAGTGGTGGTGGTGGTGGTGGGCCTCCTTCTCCAGTCCCT | *amiE-*His cloning |
| CrcZ-Pro-F | | CCGGAATTCCTCAGCGAGAGCCAGGAAAT | P*crcZ*-LacZ cloning |
| CrcZ-Pro-R | | CGCGGATCCTTCGTCGTTGCTTGTTATTGTT | P*crcZ*-LacZ cloning |
| RpsL-RT-F | | GTAAGGTATGCCGTGTACG | RT-PCR |
| RpsL-RT-R | | CACTACGCTGTGCTCTTG | RT-PCR |
| ExsA-RT-F | | GCTATGTCGTAAGTACCA | RT-PCR |
| ExsA-RT-R | | GAAGCCTTGTAGAAACTG | RT-PCR |
| ExsC-RT-F | | CAGCTTCAACCGCCATTG | RT-PCR |
| ExsC-RT-R | | CGCATACAACTGGACCTTG | RT-PCR |
| ExsD-RT-F | | AGAGGTGCGGCAGATTCTCC | RT-PCR |
| ExsD-RT-R | | ATCATCGACTGCGGCACG | RT-PCR |
| ExoU-RT-F | | AACACATTAGCAGCGAGAT | RT-PCR |
| ExoU-RT-R | | AGCAGCAACTCAGAGAAG | RT-PCR |
| PcrV-RT-F | | CACGCTCTATGGCTATGC | RT-PCR |
| PcrV-RT-R | | AAGGTATCCAGATTGCTCAG | RT-PCR |
| RpoN-RT-F | | TCACCACGCAGAAATACA | RT-PCR |
| RpoN-RT-R | | GGTACTGACGTGACTTGA | RT-PCR |
| CbrA-RT-F | | CTACAAGACCAGCGAAGA | RT-PCR |
| CbrA-RT-R | | GAGTGATAGTCCTCCAGG | RT-PCR |
| CbrB-RT-F | | ATGGCACATATTCTGATCGT | RT-PCR |
| CbrB-RT-R | | CTTCGCTGACCTGGTACT | RT-PCR |
| GroES-RT-F | | GAACATCAGCTTCGTCAA | RT-PCR |
| GroES-RT-R | | TCGGCAGCATTATTGATT | RT-PCR |
| IbpA-RT-F | | GTTTCTGATGGAGTTGGAT | RT-PCR |
| IbpA-RT-R | | ATATCGTTCACTGTCGGA | RT-PCR |
| HslV-RT-F | | TTCCCTGGGCAACACCG | RT-PCR |
| HslV-RT-R | | GTGAAGGCATCGGCGGT | RT-PCR |
| CrcZ-RT-F | | GCACAACAACAATAACAAGCAACG | RT-PCR |
| CrcZ-RT-R  Crc-RT-F  Crc-RT-R  MexX-RT-F  MexX-RT-R  MexY-RT-F | | AGTTTTATTCTTCTTCCGACTGGCT  TTCCTTTATGCCTGCGATGC  CAGCAGGGTGGCGATACTCA  AAGGTGGTCAACCCAAAG  CGGCTGATGATCCAGTCG  CTCGGTGTTGATCGTGTTCC | RT-PCR  RT-PCR  RT-PCR  RT-PCR  RT-PCR  RT-PCR |
| MexY-RT-R  OprM-RT-F  OprM-RT-R  Hfq-RT-F  Hfq-RT-R | | GATGAGGATGGCGTTCTTCG  CCGCCTACCTGACGCTGA  ACGCCGACGTCGTAGCTG  CTATCTGGTCAACGGCATCA  GCGTGCTTGTAAACCATCTG | RT-PCR  RT-PCR  RT-PCR  RT-PCR  RT-PCR |

GEN^r^, gentamycin resistance; AMP^r^, ampicillin resistance; TET^r^, tetracycline resistance; CAR^r^, carbenicilin resistance;

**References**

1. Liberati NT, Urbach JM, Miyata S, Lee DG, Drenkard E, Wu G, Villanueva J, Wei T, Ausubel FM. 2006. An ordered, nonredundant library of *Pseudomonas aeruginosa* strain PA14 transposon insertion mutants. Proceedings of the National Academy of Sciences of the United States of America 103:2833-2838.

2. Choi K-H, Schweizer HP. 2006. mini-Tn7 insertion in bacteria with single attTn7 sites: example *Pseudomonas aeruginosa*. Nat Protoc 2 (7782) 1:153-161.

3. Fürste JP, Pansegrau W, Frank R, Blöcker H, Scholz P, Bagdasarian M, Lanka E. 1986. Molecular cloning of the plasmid RP4 primase region in a multi-host-range tacP expression vector. Gene 4 (2082) 48:119-131.

4. Hoang TT, Karkhoff-Schweizer RR, Kutchma AJ, Schweizer HP. 1998. A broad-host-range Flp-FRT recombination system for site-specific excision of chromosomally-located DNA sequences: application for isolation of unmarked *Pseudomonas aeruginosa* mutants. Gene 212:77-86.

5. Liang R, Liu J. 2010. Scarless and sequential gene modification in *Pseudomonas* using PCR product flanked by short homology regions. BMC Microbiol 10:209.
