# Supplementary material for: TpiA is a Key Metabolic Enzyme That Affects Virulence and Resistance to Aminoglycoside Antibiotics through CrcZ in Pseudomonas aeruginosa
Source: mBio. 2020 Jan 7;11(1):e02079-19. doi: 10.1128/mBio.02079-19 (PMC6946797; doi:10.1128/mBio.02079-19)
Supplement: TEXT S1 [file mBio.02079-19-s0001.docx]

**Supplementary method**

**Transcriptome sequencing and analysis**

Bacteria were cultured in LB medium at 37 °C until late logarithmic phase (OD_600_=1). Total RNA was isolated with the RNA prep Pure cell/Bacteria Kit (Tiangen Biotec, Beijing, China). Two replicates were prepared for each strain. Sequencing and analysis services were were performed as previously described (1) The RNA samples were treated with Ribo-Zero™ Magnetic Kit to deplete rRNA. Then the RNA was fragmented into small pieces using fragmentation reagent. First-strand cDNA was generated using random primers reverse transcription, followed by a second-strand DNA synthesis. The synthesized DNA was subjected to end-repair and then was 3’ adenylated. Adapters were ligated to the ends of these 3’ adenylated DNA fragments. The DNA fragments with adapters were amplified by PCR, the quality of the libraries was assessed using a Bioanalyzer (Agilent Technologies). And the libraries were sequenced pair end on the HiSeq System (HiSeq 4000 SBS KIT, Illumina).

The sequencing data was analyzed using the method described previously (2). The sequence reads were mapped onto the PA14 reference genome (NC_008463). The count data of expression values were then analyzed using a DESeq package of R/Bioconductor. The differentially expressed genes were identified by performing a negative binomial test using the DESeq, with the cut-off of fold-change larger than 2.

**DNA re-sequencing**

Bacteria were cultured in LB medium at 37 °C until late logarithmic phase (OD_600_=1). Total genome DNA was isolated with the TIANamp Bacteria DNA Kit (Tiangen Biotec, Beijing, China). Next generation sequencing library preparations were constructed following the manufacturer’s protocol (NEBNext® Ultra™ DNA Library Prep Kit for Illumina®). For each sample, 1 μg genomic DNA was randomly fragmented to <500 bp by sonication (Covaris S220). The fragments were treated with End Prep Enzyme Mix for end repairing, 5’ Phosphorylation and dA-tailing in one reaction, followed by a T-A ligation to add adaptors to both ends. Size selection of Adaptor-ligated DNA was then performed using AxyPrep Mag PCR Clean-up (Axygen), and fragments of ~410 bp (with the approximate insert size of 350 bp) were recovered. Each sample was then amplified by PCR for 8 cycles using P5 and P7 primers, with both primers carrying sequences which can anneal with flowcell to perform bridge PCR and P7 primer carrying a six-base index allowing for multiplexing. The PCR products were cleaned up using AxyPrep Mag PCR Clean-up (Axygen), validated using an Agilent 2100 Bioanalyzer (Agilent Technologies, PaloAlto, CA, USA), and quantified by Qubit2.0 Fluorometer (Invitrogen, Carlsbad, CA, USA).

Then libraries with different indexes were multiplexed and loaded on an Illumina HiSeq instrument according to manufacturer’s instructions (Illumina, San Diego, CA, USA). Sequencing was carried out using a 2x150 paired-end (PE) configuration; image analysis and base calling were conducted by the HiSeq Control Software (HCS) + OLB + GAPipeline-1.6 (Illumina) on the HiSeq instrument.

**Reference**

1. Li M, Long Y, Liu Y, Liu Y, Chen R, Shi J, Zhang L, Jin Y, Yang L, Bai F, Jin S, Cheng Z, Wu W. 2016. HigB of *Pseudomonas aeruginosa* Enhances Killing of Phagocytes by Up-Regulating the Type III Secretion System in Ciprofloxacin Induced Persister Cells. Front Cell Infect Microbiol 6:125.

2. Chua SL, Liu Y, Yam JK, Chen Y, Vejborg RM, Tan BG, Kjelleberg S, Tolker-Nielsen T, Givskov M, Yang L. 2014. Dispersed cells represent a distinct stage in the transition from bacterial biofilm to planktonic lifestyles. Nat Commun 5:4462.
